# Identification of upstream miRNAs of SNAI2 and their influence on the metastasis of gastrointestinal stromal tumors

**DOI:** 10.1186/s12935-019-1006-8

**Published:** 2019-11-12

**Authors:** Jie Ding, Yu Xia, Zhaoyan Yu, Jing Wen, Zhuxue Zhang, Zhongmin Zhang, Zhenhua Liu, Zhuan Jiang, Hang Liu, Guoqing Liao

**Affiliations:** 1 Department of Gastrointestinal Surgery, Guizhou Provincial People’s Hospital, 83 East Zhongshan Rd, Guiyang, 550002 Guizhou China; 2 Department of Stomatology, Guizhou Provincial People’s Hospital, Guiyang, 550002 China; 3 Department of Pathology, Guizhou Provincial People’s Hospital, Guiyang, 550002 China; 4 Department of Gastrointestinal Surgery, Xiangya Hospital, Central South University, Changsha, 410008 China

**Keywords:** SNAI2, miRNA, Gastrointestinal stromal tumor, Metastasis

## Abstract

**Background:**

SNAI2, a member of the snail zinc finger protein family, plays an important role in the metastasis of several types of carcinoma.

**Objective:**

This study aims to investigate the upstream miRNAs of SNAI2 and their influence on the metastasis of gastrointestinal stromal tumors (GISTs).

**Methods:**

The expression levels of SNAI2, CDH1, and CDH2 in GISTs were determined by immunohistochemistry, and the correlations with their clinicopathologic characteristics were analyzed. Subsequently, the miRNAs involved in regulating SNAI2 expression were predicted by bioinformatics technique, screened by miRNA microarray tests, and verified by real-time PCR, dual luciferase reporter assay, and invasion assay. The influence of SNAI2 and miRNAs on the invasive ability of the GIST cells and the related mechanism were detected.

**Outcomes:**

SNAI2 expression significantly increased and CDH1 expression markedly decreased in the cases of GISTs with distant metastasis. Silencing of the SNAI2 gene impaired the invasiveness of GIST cells in vitro. MiR-200b-3p, miR-30c-1-3P, and miR-363-3P were verified as the upstream metastasis-associated miRNAs of SNAI2 in GISTs by miRNA microarray, real-time PCR, dual luciferase reporter assay, and invasion assay. They bound to the 3′-UTR of SNAI2, downregulated SNAI2 expression, and inhibited the invasiveness of GIST cells. SNAI2 targetedly bound to the promoter of the CDH1 gene, downregulated the expression of CDH1, and contributed to the metastasis of GISTs.

**Conclusion:**

SNAI2 and CDH1 correlated with the metastasis of GISTs, and silencing of the SNAI2 gene impaired the invasiveness of GIST cells. MiR-200b-3p, miR-30c-1-3P, and miR-363-3P contribute to the metastasis of GISTs in vitro by mediating the SNAI2/CDH1 axis. SNAI2 may be a potential target for the treatment of GISTs in the future.

## Background

Gastrointestinal stromal tumors (GISTs) are the most common mesenchymal malignancies in the digestive tract [1], and over 90% of GISTs are derived from c-Kit and PDGFRA gene mutations [2, 3]. GISTs were first described by Mazur and Clark [4].The independent division of GISTs has exerted a considerable influence on their diagnosis and treatment. Although the integration of surgery and molecular-targeted therapy significantly improved the therapeutic effect of GISTs, more than 30% of the patients relapsed within 5 years and eventually died from this disease [5, 6].To date, the molecular mechanisms underlying GIST metastasis remain to be elucidated.

SNAI2, a member of the snail zinc finger protein family, binds to E-box motifs, represses CDH1 transcription, and contributes to the metastasis of various [7–10]. The transcriptional factor SNAI2 correlates closely with the metastasis of GISTs [11], and silencing of the SNAI2 gene impairs the invasiveness of GIST cells in vitro. However, the mechanism by which SNAI2 regulates the metastasis of GISTs remains unclear. In malignant tumors of epithelial origin, SNAI2 promotes tumor cell metastasis through epithelial–mesenchymal transition (EMT) [12–16]. Lysine-specific demethylase 1 (LSD1) is essential in SNAI2-mediated transcriptional inhibition during EMT; in the absence of LSD1, SNAI2 fails to repress CDH-1 transcription [17]. SNAI2 is not specific for malignant tumors of epithelial origin; it also contributes to the metastasis of tumors of mesenchymal origin, such as osteosarcoma [18], chondrosarcoma [19], Ewing sarcoma [20], leiomyosarcoma [21], rhabdomyosarcoma [22], synovial sarcomas [23], GISTs [11], fibrosarcoma [24], and even Kaposi sarcoma [25, 26].

MicroRNA (MiRNA), an endogenous non-encoded RNA with a length of about 19–25 nucleotides, can interact with its targets in the 3′-UTR of transcripts and result in either mRNA degradation or translation inhibition in a sequence-dependent manner [27–30]. MiRNAs may be an effective molecular biomarker for tumor diagnosis and treatment [31]. MiRNAs could directly target genes and play a central role in EMT, modulating the metastatic process [32, 33]. Many miRNAs target SNAI2 and regulate the metastasis of cancer [34–36]. However, the miRNAs targeting the expression of SNAI2 in GISTs remain undetermined. In the present study, we identified the miRNAs that regulate SNAI2 expression and investigates the molecular mechanisms by which they influence GIST metastasis. Furthermore, we assessed the feasibility of using exogenous miRNAs to inhibit the invasion and metastasis of GIST cells. This study could serve as a basis for developing novel treatments for GISTs.

## Methods

### Patients and specimens

The archival formaldehyde-fixed and paraffin-embedded tumor tissue of 78 GIST specimens surgically removed from 2004 to 2007 were retrieved from the Department of Pathology, Xiangya Hospital, Central South University. No new adjuvant therapy was administered before surgery. Patient age ranged from 28 to 87 years old (median 51), and 48 were male and 30 were female. The primary tumor sites of the GIST specimens include the stomach (*n* = 47), small intestine (*n* = 26), and colorectum (*n* = 5). In accordance with the modified NIH consensus criteria [37], 5 very-low-risk, 20 low-risk, 22 intermediate-risk, and 31 high-risk cases were identified (Table 1). This research was conducted with the approval of the ethics committee of Guizhou Provincial People’s Hospital, China, and the ethics committee of Xiangya Hospital, Central South University. Documented informed consents were obtained from all patients.

**Table 1:** Correlation between expression of SNAI2, E-cadherin, N-cadherin and clinicopathological characteristics of GIST patients

Item	n	SNAI2 (+)		χ^2^	*P* value	E-cad (+)		χ^2^	*P* value	N-cad (+)		χ^2^	*P* value
		n	%			n	%			n	%		
Gender													
Male	48	28	58.3			21	43.8			39	81.3		
Female	30	14	46.7	1.01	0.32	7	23.3	3.34	0.07	20	66.7	2.13	0.14
Age													
< 50 years	40	24	60.0			13	32.5			29	72.5		
> 50 years	38	18	47.4	1.25	0.26	15	39.5	0.41	0.52	30	78.9	0.44	0.51
Position													
Stomach	47	24	51.1			15	31.9			35	74.5		
Intestine	26	15	57.7			10	38.5			21	80.8		
Colorectum	5	3	60	0.38	0.83	3	60	1.66	0.44	3	60	1.07	0.59
Risk category													
Very low	5	2	40			2	40			4	80.0		
Low	20	11	55			9	45			16	80.0		
Mid	22	9	40.9			9	40.9			14	63.6		
High	31	20	64.5	3.30	0.348	8	25.8	2.37	0.50	25	80.6	2.4	0.49
Local invasion													
Yes	41	25	61			16	39.0			29	70.7		
No	37	17	45.9	1.77	0.18	12	32.4	0.37	0.54	30	81.1	1.13	0.29
Distant metastasis													
Yes	24	18	75			4	16.7			20	83.3		
No	54	24	4.4	6.24	0.01	24	44.4	5.57	0.02	39	72.2	1.11	0.29

### Cell lines and cell culture

Both GIST882 and GIST-T1 were established from untreated human metastatic GISTs. GIST882 harbors a homozygous exon 13 missense mutation [38], and GIST-T1 has a heterogenic 57-bp deletion in exon 11 to produce a mutated c-KIT [39]. GIST882 cells were maintained in RPMI1640 supplemented with 10% fetal bovine serum (FBS), and GIST-T1 cells were cultured in Dulbecco’s modified Eagle’s medium supplemented with 10% FBS. GIST cells were grown in cell culture flasks at 37 °C in a humidified atmosphere of 95% air and 5% CO_2_.

### miRNA target prediction

Targetscan v7.1 was used to predict miRNA target.

### miRNAs microarray

Human microRNA microarrays (HmiOA7.0, PhalanxBio Inc.) were used in GIST samples. The microarray contains probes for 2003 human microRNAs from Sanger miRBase release 19.0. Total RNA (100 ng) derived from GIST samples was labelled with Cy5 or Cy3. Microarray slides were scanned by DNA Microarray Scanner G2565B (Agilent Technology). Labeling and hybridization were performed in accordance with the protocols in the PhalanxBio miRNA microarray system. The microarray image information was converted into spot intensity values using Feature Extraction Software. The signal after background subtraction was exported directly into the GeneSpring GX10 software (Agilent Technologies, Santa Clara, CA).

### Transient transfection of miRNA mimics and inhibitors

GIST cells (5 × 10^4^) were seeded in 24-well plates 24 h before transfection. The medium was replaced with antibiotics-free media 6 h before transfection. Selected miRNA mimics, inhibitors, and a negative control (from Sigma-Aldrich) were transfected into GIST cells using Lipofectamine™ 3000 following the Sigma-Aldrich transfection protocol. After 24 h, the cells were split into two 24-well plates in antibiotics-containing media and cultured for an additional 48 h. The cells were then washed twice with PBS and lysed in TRIzol reagent (Invitrogen). The sequences of the miRNA mimics and inhibitors used in this study are as follows:

**Table:** 

miRNA	Sense (5′–3′)
miR-30c-1-3p mimics	CUGGGAGAGGGUUGUUUACUCC
miR-30c-1-3p inhibitors	GGAGUAAACAACCCUCUCCCAG
miR-363-3p mimics	AAUUGCACGGUAUCCAUCUGUA
miR-363-3p inhibitors	UACAGAUGGAUACCGUGCAAUU
mir-1-3p mimics	UGGAAUGUAAAGAAGUAUGUAU
mir-1-3p inhibitors	AUACAUACUUCUUUACAUUCCA
mir-375 mimics	UUUGUUCGUUCGGCUCGCGUGA
mir-375 inhibitors	UCACGCGAGCCGAACGAACAAA
mir-32-3p mimics	CAAUUUAGUGUGUGUGAUAUUU
mir-32-3p inhibitors	AAAUAUCACACACACUAAAUUG
Mimics NC	UUGUACUACACAAAAGUACUG
Inhibitor NC	CAGUACUUUUGUGUAGUACAA

### Transient transfection of siRNA or cDNA

Cells (5 × 10^4^) were seeded in 6-well plates and then incubated for 2–4 days in standard medium in the presence of 10–20 nmol/L siRNA or cDNA directed against target genes. Cells were transfected using Lipofectamine 3000 (Invitrogen) in accordance with the manufacturer’s instructions. Cells were transfected with a scrambled siRNA as a control and untreated cells as a blank control. After 24 h, transfection efficiency was assessed as GFP fluorescence under a fluorescence microscope. Human SNAI2 cDNA was produced by PCR amplification of reverse-transcribed products of total RNA from GIST882 cells by using the specific primers, (F: 5′-ATGCCGCGCTCCTTCCTGGT-3′, R: 5′-TCAGTGTGCTACACAGCAGCC-3′).

The siRNA sequences used in this study were as follows:

**Table:** 

siRNA	Sense (5′–3′)	Antisense (5′–3′)
SNAI2 (#1)	CGUAUCUCUAUGAGAGUUATT	UAACUCUCAUAGAGAUACGTT
SNAI2 (#2)	CAUUCUGAUGUAAAGAAAUTT	AUUUCUUUACAUCAGAAUGTT
SNAI2 (#3)	CAUGGAAUUCAUGUGUUUATT	UAAACACAUGAAUUCCAUGTT
CDH1 (#1)	CCUCGACACCCGAUUCAAATT	UUUGAAUCGGGUGUCGAGGTT
CDH1 (#2)	CCGAUCAGAAUGACAACAATT	UUGUUGUCAUUCUGAUCGGTT
CDH1 (#3)	GGUUCAAGCUGCUGACCUUTT	AAGGUCAGCAGCUUGAACCTT
CDH2 (#1)	GUGCAGUCUUAUCGAAGGATT	UCCUUCGAUAAGACUGCACTT
CDH2 (#2)	AAGUACAAUAUGAGAGCAGTT	CUGCUCUCAUAUUGUACUUTT
CDH2 (#3)	UGGCAUGGUGUAUGCCGUGTT	CACGGCAUACACCAUGCCATT
Control siRNA	UUCUCCGAACGUGUCACGUTT	ACGUGACACGUUCGGAGAATT

### Immunohistochemical staining

Primary antibodies were directed toward SNAI2 (rabbit monoclonal, 1:200; R&D Systems, Minneapolis, MN, USA), CDH1 (rabbit polyclonal, 1:200; R&D Systems), and CDH2 (rabbit polyclonal, 1:100; R&D Systems). Serial sections of 5 µm were cut from the tissue blocks, deparaffinized in xylene, and hydrated in a graded series of alcohol. Staining was then performed using the EnVision + anti-rabbit system (Dako Corporation, Carpinteria, CA, USA). Negative control staining was carried out by substituting nonimmune rabbit and phosphate-buffered saline for the primary antibodies.

### Evaluation of immunohistochemical staining results

The evaluations were performed by two pathologists who were unaware of the patients’ information. In cases of disagreement, another review and discussion was performed by both pathologists to obtain a consensus. The two-way scoring system of staining intensity and staining extent was used to analyze the immunohistochemical staining results. Staining intensity was graded as follows: negative (0), weak (1), moderate (2), and strong (3). Staining extent was rated according to the percentage of positive cells. Samples with no stained tumor cells were rated as 0, those with < 25% of stained tumor cells as 1, those with 25–50% as 2, and those with > 50% as 3. The results of staining intensity and extent produced an overall staining score. An overall score of 0 was marked as negative (−), 1–2 as weak (+), 3–4 as moderate (++), and 5–6 as strong (+++).

### Western blot

Protein lysates were extracted from cells and blotted as described previously [40]. The membranes were incubated overnight using the following antibodies and dilutions: SNAI2, 1:1000; CDH-1, 1:2000; CDH2, 1:2000; and GAPDH, 1:2000.

### Real-time PCR

The total RNA was extracted using Trizol reagent (Invitrogen, CA, USA). cDNA synthesis was generated using 1 µg of total RNA with an iScript cDNA Synthesis Kit (Bio-Rad, CA, USA). The reaction mixture was initially denatured for 10 min at 95 °C, followed by 40 PCR cycles of a denaturing step (95 °C for 15 s) and a primer annealing/extension step (60 °C for 60 s). The expression values were normalized to the geometric mean of GAPDH. The primers used in this study are as follows:

**Table:** 

miRNA	F (5′–3′)	R (5′–3′)
hsa-miR-1-3p	GGGTGGAATGTAAAGAAGT	TTTGGCACTAGCACATT
hsa-miR-375	GTTTTGTTCGTTCGGCTC	TTTGGCACTAGCACATT
hsa-miR-32-3p	GGGCAATTTAGTGTGTGTG	TTTGGCACTAGCACATT
hsa-miR-30c-1-3p	TTCTGGGAGAGGGTTGT	TTTGGCACTAGCACATT
hsa-miR-200c-3p	GGTAATACTGCCGGGTAAT	TTTGGCACTAGCACATT
hsa-miR-200b-3p	GGGTAATACTGCCTGGTAA	TTTGGCACTAGCACATT
hsa-miR-363-3p	GGATTGCACGGTATCCA	TTTGGCACTAGCACATT
hsa-miR-182-5p	GGTTTGGCAATGGTAGAACT	TTTGGCACTAGCACATT
GAPDH	TACTAGCGGTTTTACGGGCG	TCGAACAGGAGGAGCAGAGAGCGA

### ChIP

Chromatin immunoprecipitation (ChIP) analysis was performed using Protein A and Protein G Dynabeads (Invitrogen) as previously reported [41]. Cells were exposed to 1% formaldehyde to crosslink proteins, and 1.0 × 10^7^ cells were used for each ChIP assay. Quantitative ChIP was performed using qPCR on the ABI PRISM 7900 real-time PCR detection system (Applied Biosystems). Primer sequences for the qPCR of promoters of target genes for ChIP were as follows: CDH1,F: 5′-AGTCCCACAACAGCATAGGG-3′, R: 5′-TTCTGAACTCAGGCGATCCT-3′; CDH2, F: 5′-GGGTAAGAACAAGCACTTCTGA-3′, R: 5′-TACTGTTGCTGGCTAGGCTT-3′; GAPDH, F: 5′-TACTAGCGGTTTTACGGGCG-3′, R: 5′-TCGAACAGGAGGAGCAGAGAGCGA-3′. Sheared genomic DNA was used as a positive control (input) and for the normalization of DNA immunoprecipitated by SNAI2.

### Transwell invasion assay

Invasion assays were performed by using BD BioCoat™ Matrigel Invasion Chambers. In brief, cells in the log growth phase were trypsinized and suspended in serum-free media (with 0.04%BSA) at a density of 4 × 10^5^ cells/mL. Designated control or treated suspended cells (0.5 mL) were added to each migration or invasion chamber and incubated at 37 °C for 22 h. In addition, 25 ng/mL HGF (0.75 mL) was added to the lower well of each companion plate to attract cells from migration or invasion chamber plate inserts at the top of the companion plate. Cells that invaded or migrated the lower wells were then fixed and stained with 0.5% Toluidine Blue for 15 min at 37 °C and washed twice with TBS. The stained cells were counted under an inverted microscope (5 fields per membrane). All experiments were conducted at least three times in triplicate.

### Scratch wound-healing assay

GIST cells were cultured until they reached 90% confluence in 25 mm dishes. Subsequently, scratches were generated using a sterile 20 μL pipette tip prior to cells being treated with SNAI2 siRNA or NC siRNA for 48 h. The border of the denuded area was immediately marked with a fine line, and cells were incubated in RPMI1640 supplemented with 10% FBS. Images of the cell cultures were captured at 48 h using an inverted phase contrast microscope. Assays were performed in triplicate.

### Luciferase reporter assay

The 1130 nt SNAI2 3′-UTR (GenBank ID: NM_003068) was cloned into the multiple cloning site of the pGL3 dual-luciferase miRNA target expression vector (Promega, Madison, WI). SNAI2 was co-transfected with 1 μg of constructed plasmids and 100 nM of miRNA mimics and the negative control using Lipofectamine™ 3000 (Invitrogen, Carlsbad, CA). Empty vector was used as a blank control. After 48 h of transfection, cells were harvested to measure luciferase activity using the Luciferase Assay System Kit (Promega, E1500) in accordance with the manufacturer’s instructions.

### Statistical analysis

Statistical analysis was performed using SPSS V.17.0 software. Differences in the expression levels of SNAI2, CDH1, and CDH2 among different clinicopathological characteristics were analyzed by χ^2^ test. All in vitro assays were repeated three times to provide biological replicates. Statistical comparisons were performed using two-tailed student’s T-test or two-way ANOVA as appropriate. Statistical significance was considered at P < 0.05.

## Results

### SNAI2 correlated closely with the metastasis of GISTs, and silencing of the SNAI2 gene impaired the invasiveness of GIST cells in vitro

**Fig. 1:**
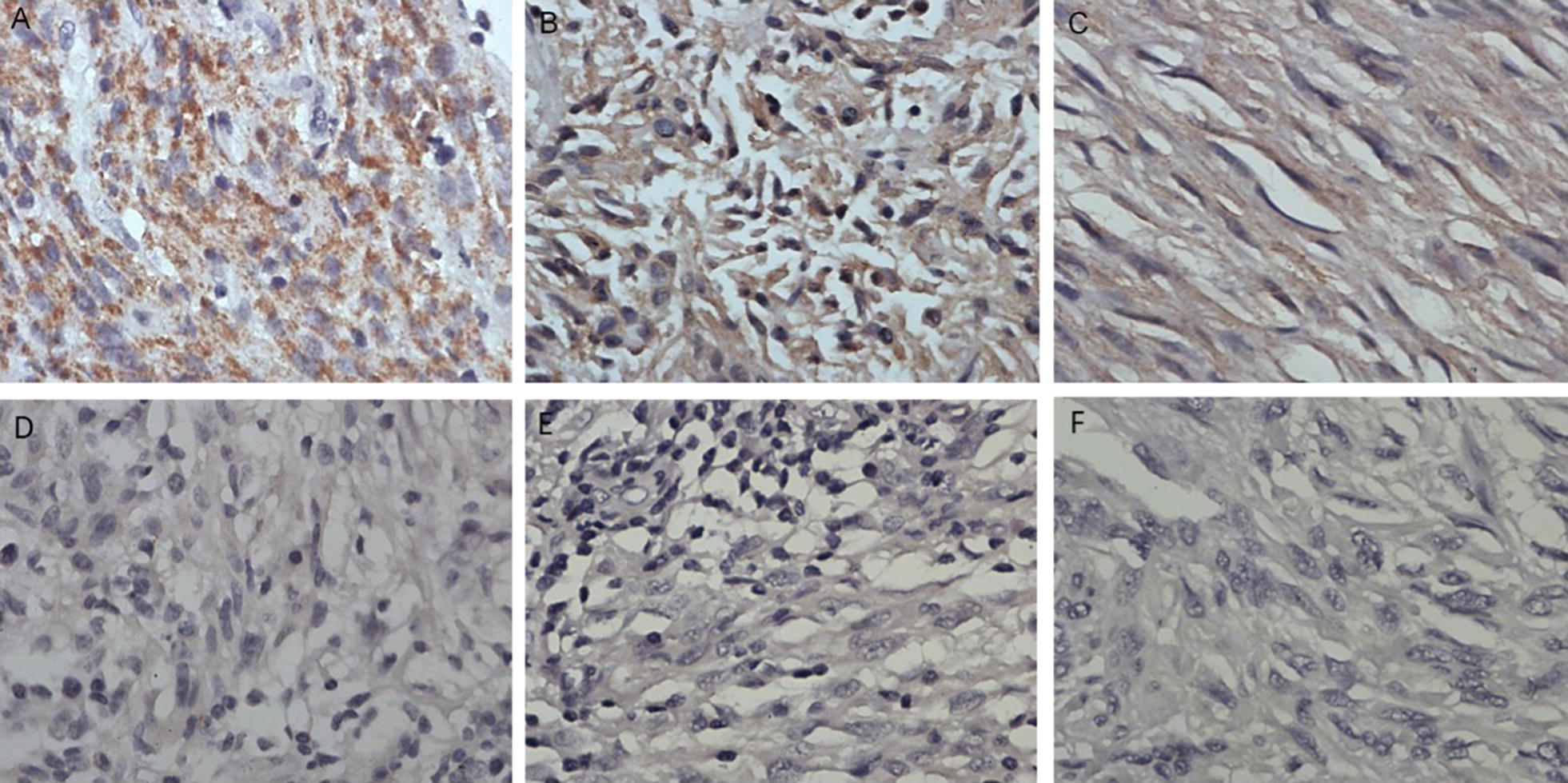
Immumohistochemical staining of SNAI2, CDH1 and CDH2 staining in GIST specimens. (**A**, **D**) Representative positive and negative staining of SNAI2 in GISTs specimens. (**B**, **E**) Representative positive and negative staining of CDH1 in GISTs specimens. (**C**, **F**) Representative positive and negative staining of CDH2 in GISTs specimens. The positive expression of SNAI2, CDH1 and CD2 in GISTs were 53.8%, 35.9% and 75.6% respectively

Seventy-eight GIST specimens removed by surgery from 2004 to 2007 were obtained in Xiangya Hospital, Central South University. The expression levels of SNAI2, CDH1, and CDH2 in the GISTs were determined by immunohistochemistry to determine whether the expression levels of SNAI2, CDH1, and CDH2 correlate with the clinicopathological characteristics of GIST patients. SNAI2 protein was mainly located in the cytoplasm of GIST cells, with a few cells showing localization in the nucleus. CDH1 and CDH2 proteins were localized in the cytomembrane and cytoplasm of GIST cells (Fig. 1). As shown in Table 1, the positive expression of SNAI2, CDH1, and CD2 in GISTs were 53.8%, 35.9%, and 75.6%, respectively. SNAI2 expression significantly increased in the cases of GISTs with metastasis (P < 0.05). CDH1 expression markedly decreased in the cases with distant metastasis (P < 0.05). However, the expression of CDH2 did not significantly change among the different clinicopathological characteristics (P > 0.05).

**Fig. 2:**
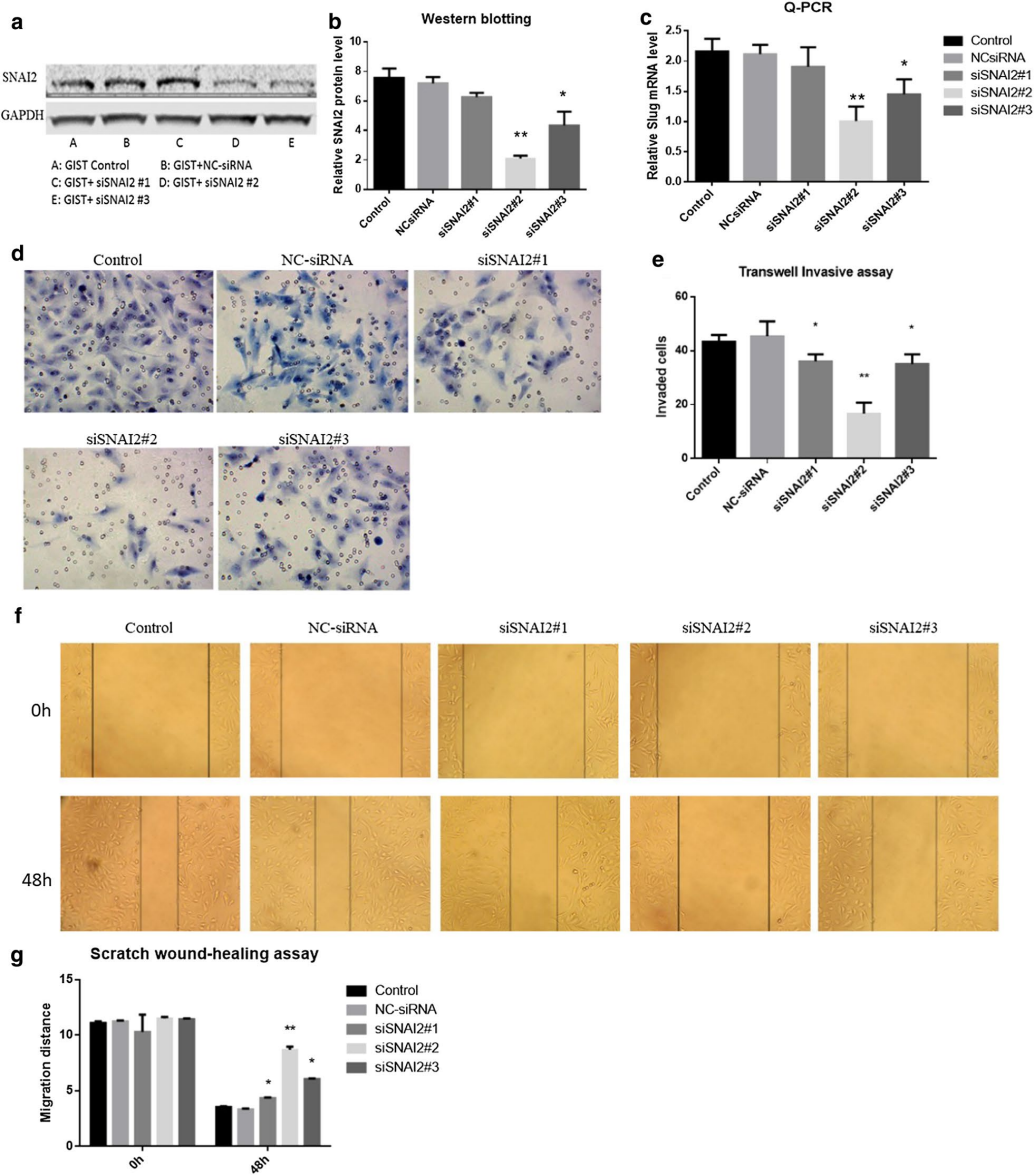
The knockdown effects of siRNAs on SNAI2. **a**, **b** The knockdown effect of siSNAI2#2 was more efficient than the other two siRNAs at protein level. **c** The knockdown effect of siSNAI2#2 was more efficient than the other two siRNAs at mRNA level. **d**–**g** The invasiveness of GIST cells was downregulated after SNAI2 siRNA transfection, especially in siSNAI2#2 group

We conducted a SNAI2 gene silencing experiment to investigate the role of SNAI2 in the metastasis of GIST cells. First, three siRNAs targeting SNAI2 (siSNAI2#1, siSNAI2#2, and siSNAI2#3) were transfected to GIST cells to detect gene-silencing efficiency. The knockdown effect of siSNAI2#2 was better than that of the two other siRNAs at the mRNA and protein levels (Fig. 2). Using the siSNAI2#2, we performed Transwell Invasion Assay and Scratch wound-healing assay in GIST cell lines and found a significant inhibition of invasion by siRNA (Fig. 2). However, the mechanism by which SNAI2 regulates the metastasis of GISTs remains unclear.

### Prediction and screening of the upstream metastasis-associated miRNAs of SNAI2 in GISTs

Targetscan is a server for predicting the target genes of microRNAs (miRNAs). It was used to predict the upstream miRNAs of SNAI2. Results showed that 425 miRNAs may target SNAI2 (Additional file 1). Then, miRNA microarray was used to detect the differentially expressed miRNAs between three high-SNAI2-level GISTs (+++) and three low-SNAI2-level GISTs (−). Results showed that 339 miRNAs were upregulated and 85 miRNAs were downregulated in the high-SNAI2-level GISTs compared with the low-SNAI2-level GISTs (Additional file 2, Fig. 3).

**Fig. 3:**
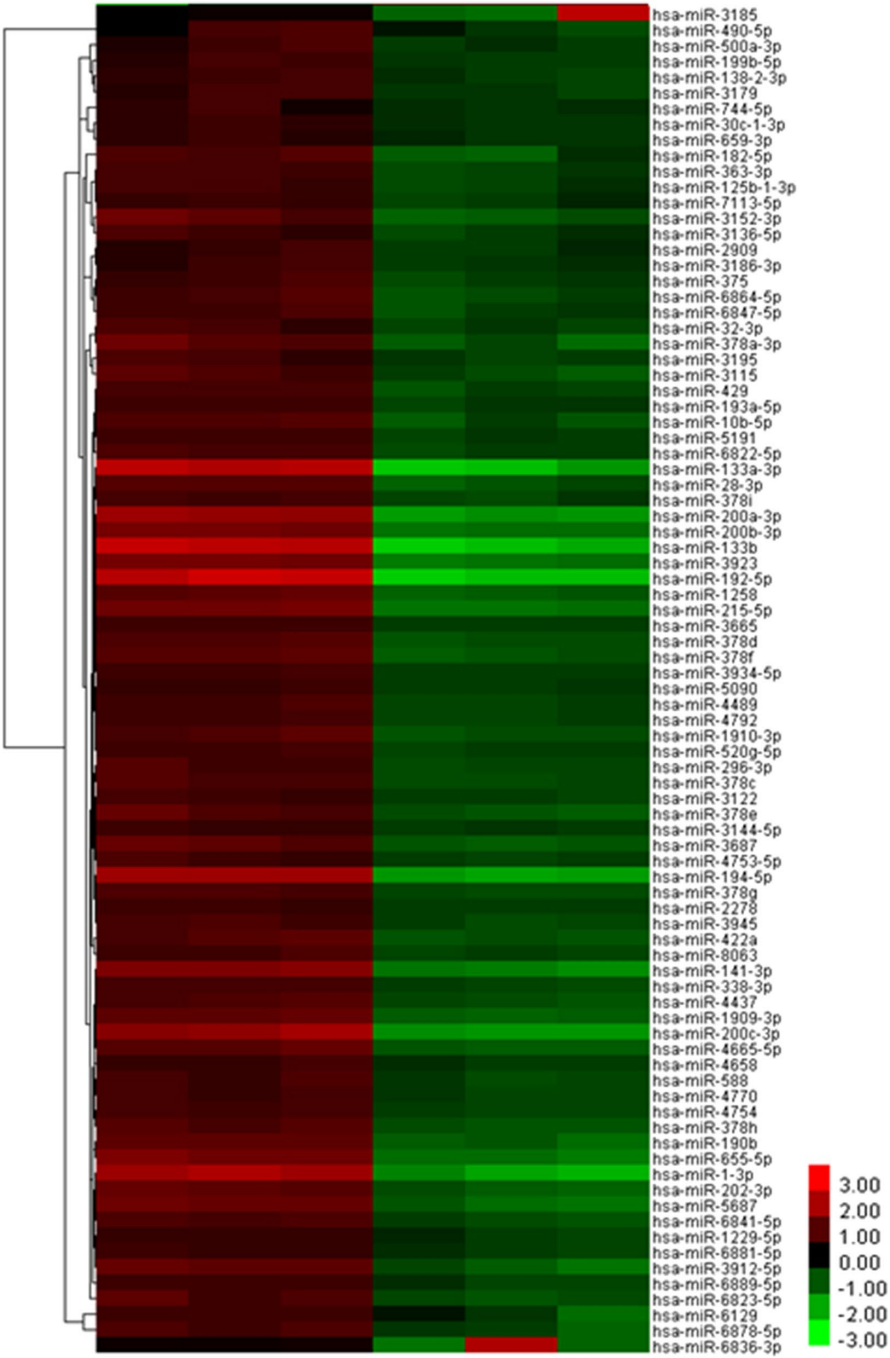
Unsupervised hierarchical clustering analysis of miRNA expression profiles from GISTs specimen:85 miRNAs were downregulated in high SNAI2 level GISTs compared with low SNAI2 level GISTs

**Fig. 4:**
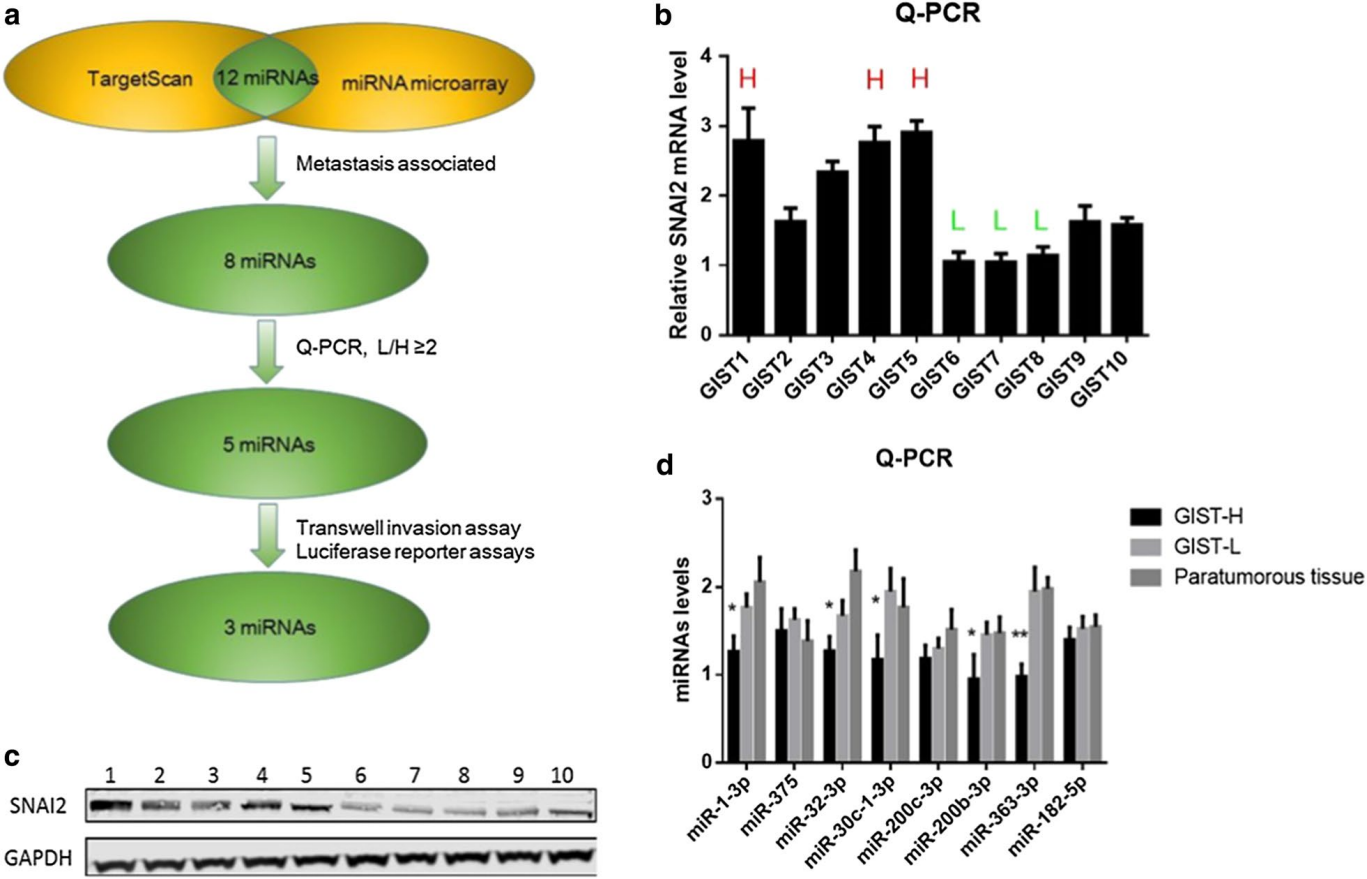
Prediction and screening of the upstream metastasis-associated miRNAs of SNAI2 in GISTs. **a** Flowchart of confirmation of the upstream metastasis-associated miRNAs of SNAI2 in GISTs. **b**, **c** The mRNA and protein levels of SNAI2 in 10 GISTs tissue, 3 high SNAI2 level GISTs and 3 low SNAI2 level GISTs were selected for the miRNA microarray. **d** Eight possible metastasis-associated miRNAs levels in GISTs. The miRNAs levels of five miRNAs including miRNA-1-3P, miRNA-200b-3p, miRNA-32-3P, miRNA-30c-1-3P and miRNA-363-3P were significantly lower in high SNAI2 level GISTs group compared with low SNAI2 level GISTs group

The results of TargetScan and miRNA microarray were combined to evaluate the upstream miRNAs of SNAI2 in GISTs. Twelve miRNAs were selected as the possible upstream miRNAs of SNAI2. Among which, the following eight miRNAs were associated with cancer metastasis: miR-1-3p, miR-375, miR-32-3p, miR-200b-3p, miR-200c-3p, miR-30c-1-3p, miR-363-3p, and miR-182-5p (Fig. 4).

### Confirmation of the upstream metastasis-associated miRNAs of SNAI2 in GISTs

**Fig. 5:**
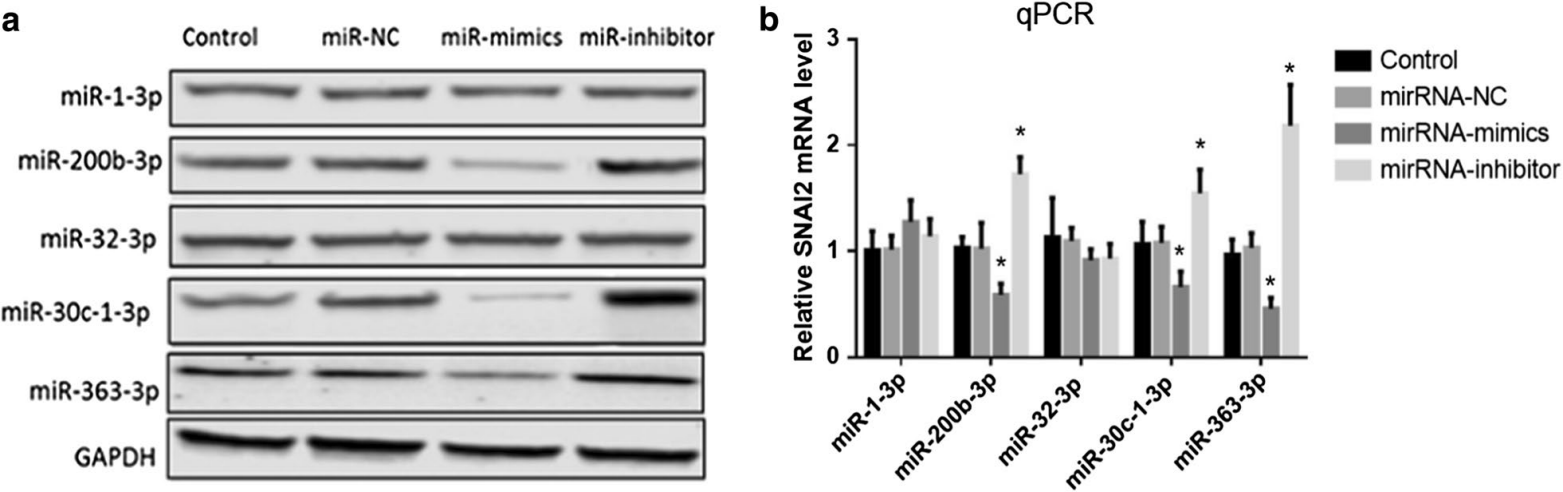
SNAI2 levels after transfection of miRNA mimics and inhibitors. **a**, **b** MiR-200b-3p, miR-30c-1-3P and miR-363-3P could downregulate the SNAI2 in both protein and mRNA level

**Fig. 6:**
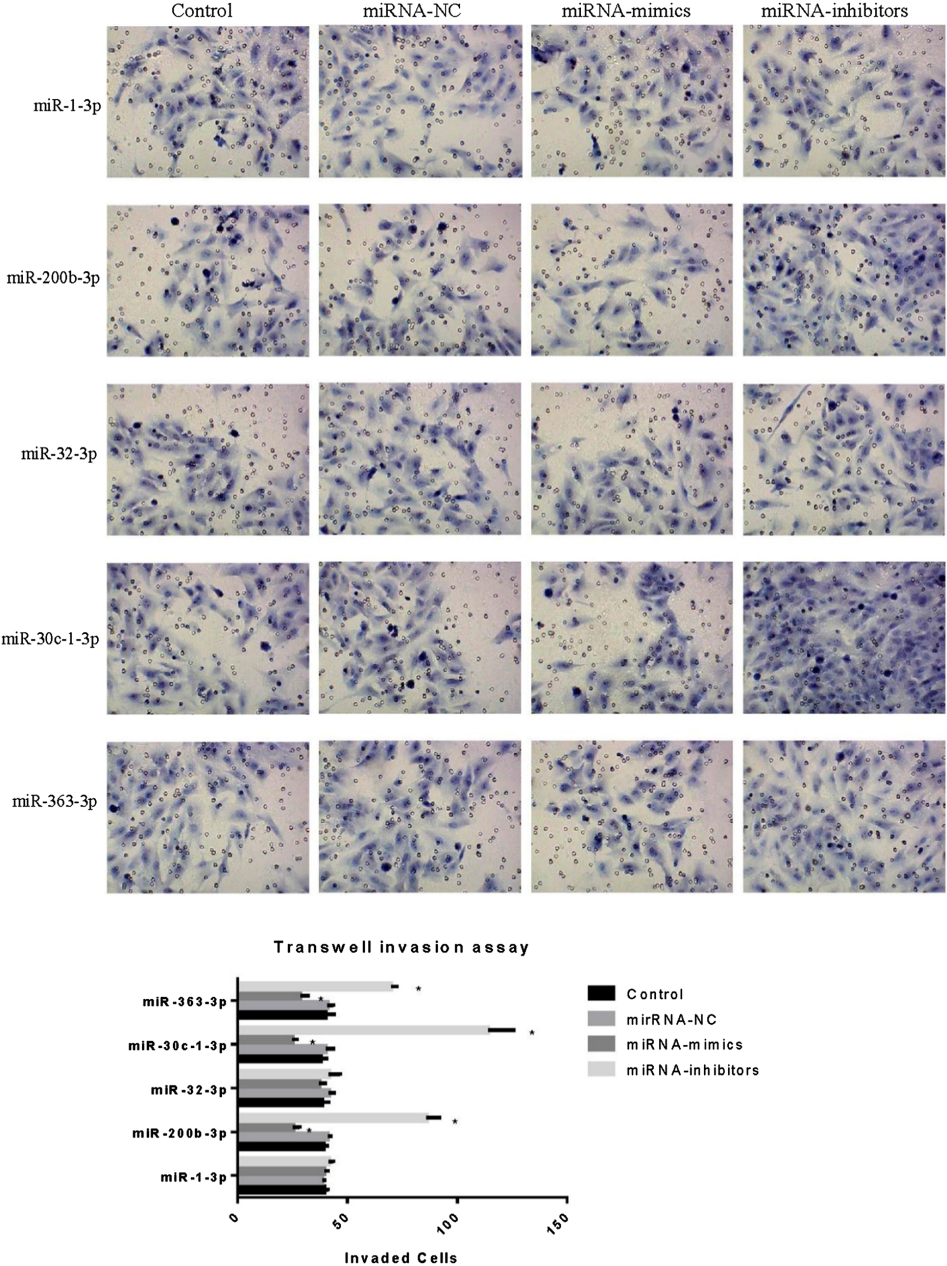
The invasiveness of GIST cells after treated with miRNA-mimics and miRNA-inhibitors. The invasiveness of GISTs cells were downregulated by miRNAs mimics and upregulated by inhibitors of 3 miRNAs, miR-200b-3p, miR-30c-1-3P and miR-363-3P

Real-time PCR was used to detect the levels of the eight possible metastasis-associated miRNAs in GISTs. According to the principle of L/H ≥ 2 (miRNA concentration in low SNAI2 level GISTs/miRNA concentration in high SNAI2 level GISTs), five miRNAs (miRNA-1-3P, miRNA-200b-3p, miRNA-32-3P, miRNA-30c-1-3P, and miRNA-363-3P) were selected as the candidate upstream miRNAs of SNAI2 (Fig. 4). Transfection of miRNA mimics and inhibitors was used to further confirm the upstream miRNAs of SNAI2. Results showed that three miRNAs, namely, miR-200b-3p, miR-30c-1-3P, and miR-363-3P, could downregulate the SNAI2 transcription (Fig. 5). Then, Transwell invasion assay was used to detect the influence of miRNAs on the invasive ability of GIST cells. Interestingly, the invasiveness of the GIST cells was downregulated by miRNA mimics and upregulated by inhibitors of three miRNAs, namely, miR-200b-3p, miR-30c-1-3P, and miR-363-3P (Fig. 6).

### MiR-200b-3p, miR-30c-1-3P, and miR-363-3P targetedly bind to the 3′-UTR of SNAI2 and downregulate SNAI2 expression

**Fig. 7:**
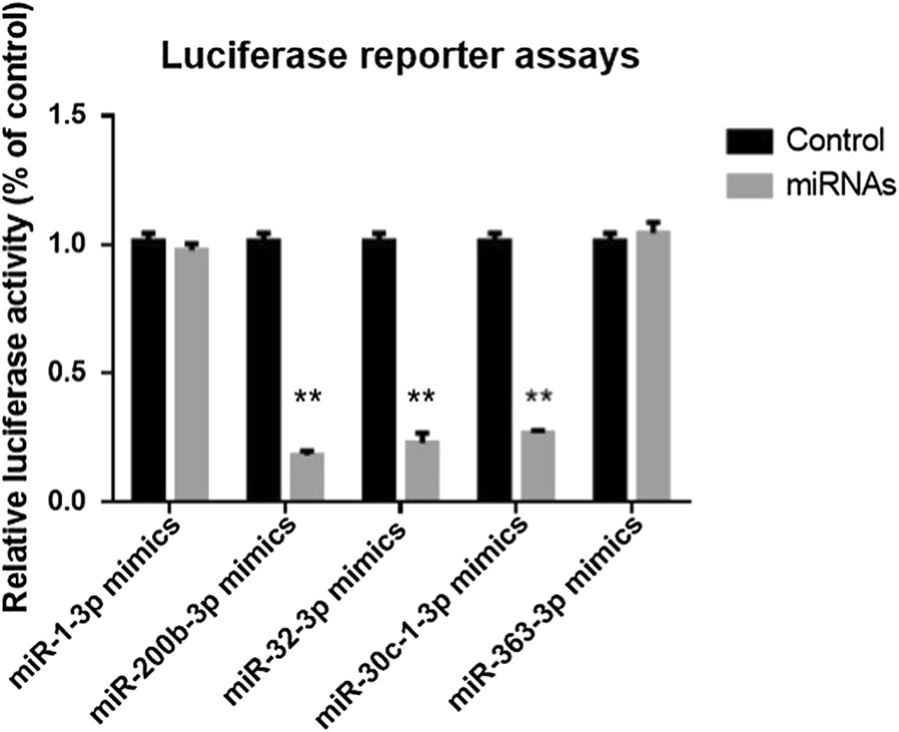
Luciferase reporter assays. Transfection of miRNA mimics of miR-200b-3p, miR-30c-1-3P, miR-363-3P resulted in a decrease in luciferase signal to from 73 to 83% of that in the negative control

Luciferase reporter assays were performed to detect the interaction of miRNAs and their targeting sequence in the 3′-UTR of SNAI2 mRNA. Five candidate miRNAs, Mimics NC were transfected into HEK293T cells that stably express a luciferase reporter containing the 3′-UTR of SNAI2 mRNA. As shown in Fig. 7, co-transfection of pGL3-SNAI2-3′-UTR and the miRNA mimics led to a downregulation of luciferase signal from 73 to 83% of that in the control group, which confirmed the direct binding of miR-200b-3p, miR-30c-1-3P, and miR-363-3P to the SNAI2 3′-UTR.

### Silencing SNAI2 led to the upregulation of CDH1 expression and downregulated the invasiveness of GIST cells

**Fig. 8:**
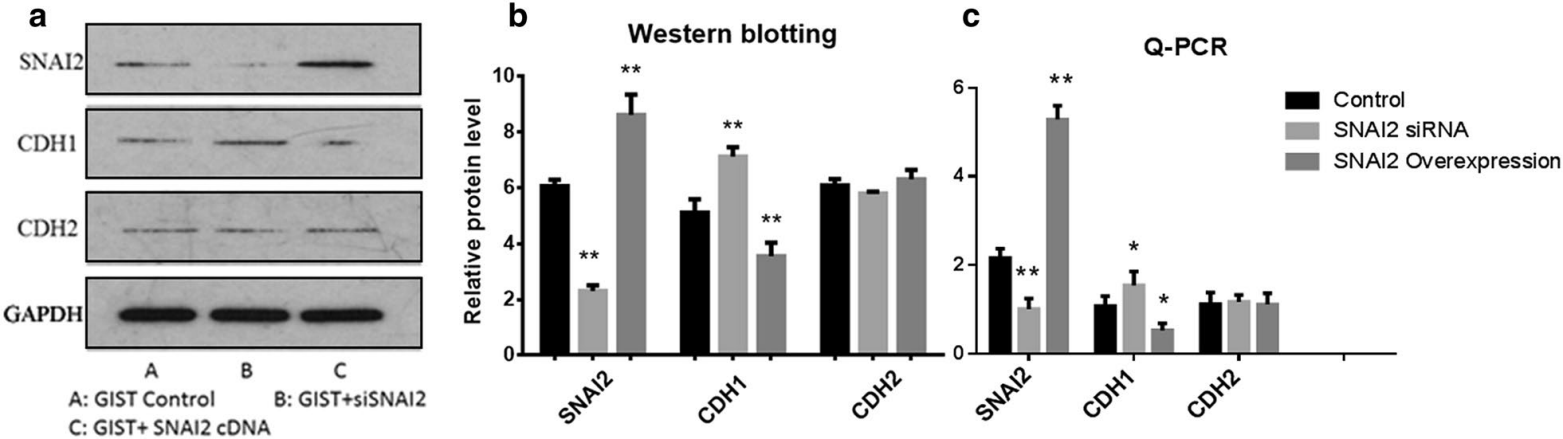
Knockdown and overexpression experiment of SNAI2. (**a**–**b**) CDH1 protein was upregulated in the SNAI2 knockdown group and downregulated in the SNAI2 overexpression group. However, CDH2 protein remained invariable. (**c**) CDH1 mRNA was upregulated in the SNAI2 knockdown group and downregulated in the SNAI2 overexpression group. However, CDH2 mRNA remained invariable

**Fig. 9:**
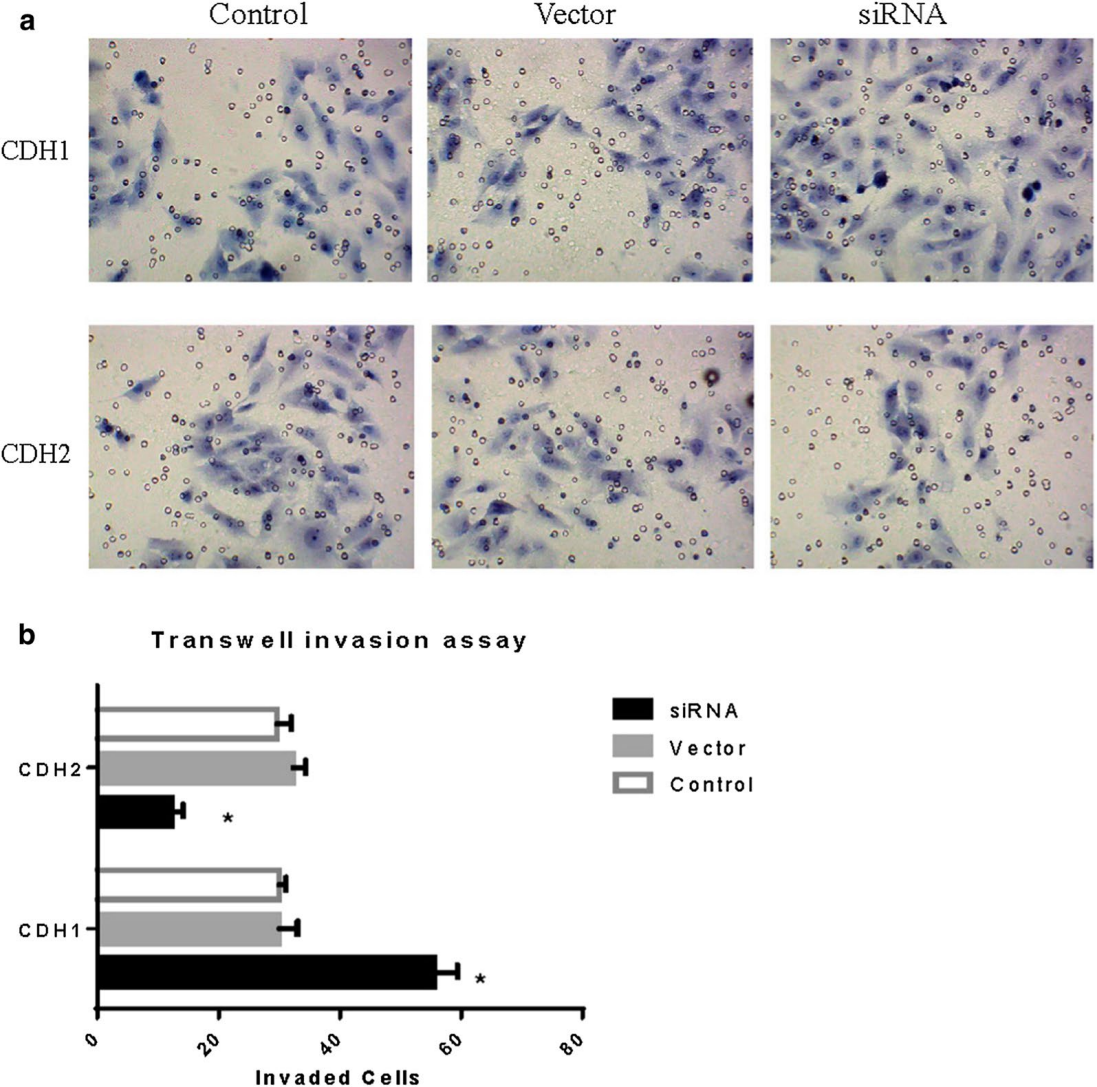
The invasiveness of GIST cells after knock down of CDH1 and CDH2. **a**, **b** The invasive ability of GIST cells was upregulated by CDH1 inhibition, and downregulated by CDH2 inhibition

SNAI2 acts as an inhibitory transcription factor that binds to E-box motifs of CDH1 and represses its transcription in cancer [9]. However, the mechanism by which SNAI2 affects the invasive ability of GIST cells is unclear. We performed a knockdown and overexpression experiment of SNAI2 to further investigate whether CDH1 and CDH2 are the downstream target genes of SNAI2 in GIST cells. At 48 h after transfection, the expression of CDH1 was upregulated in the SNAI2 knockdown group and downregulated in the SNAI2 overexpression group. However, the expression of CDH2 remained invariable (Fig. 8). Transwell Invasion Assay was used to investigate the invasiveness following the knockdown of CDH1 and CDH2 of GIST cells. Results showed that the invasive ability of the GIST cells were upregulated by CDH1 inhibition and downregulated by CDH2 inhibition (Fig. 9).

### SNAI2 targetedly binds to the promoter of the CDH1 gene, downregulates the expression of CDH1, and contributes to the metastasis of GISTs

**Fig. 10:**
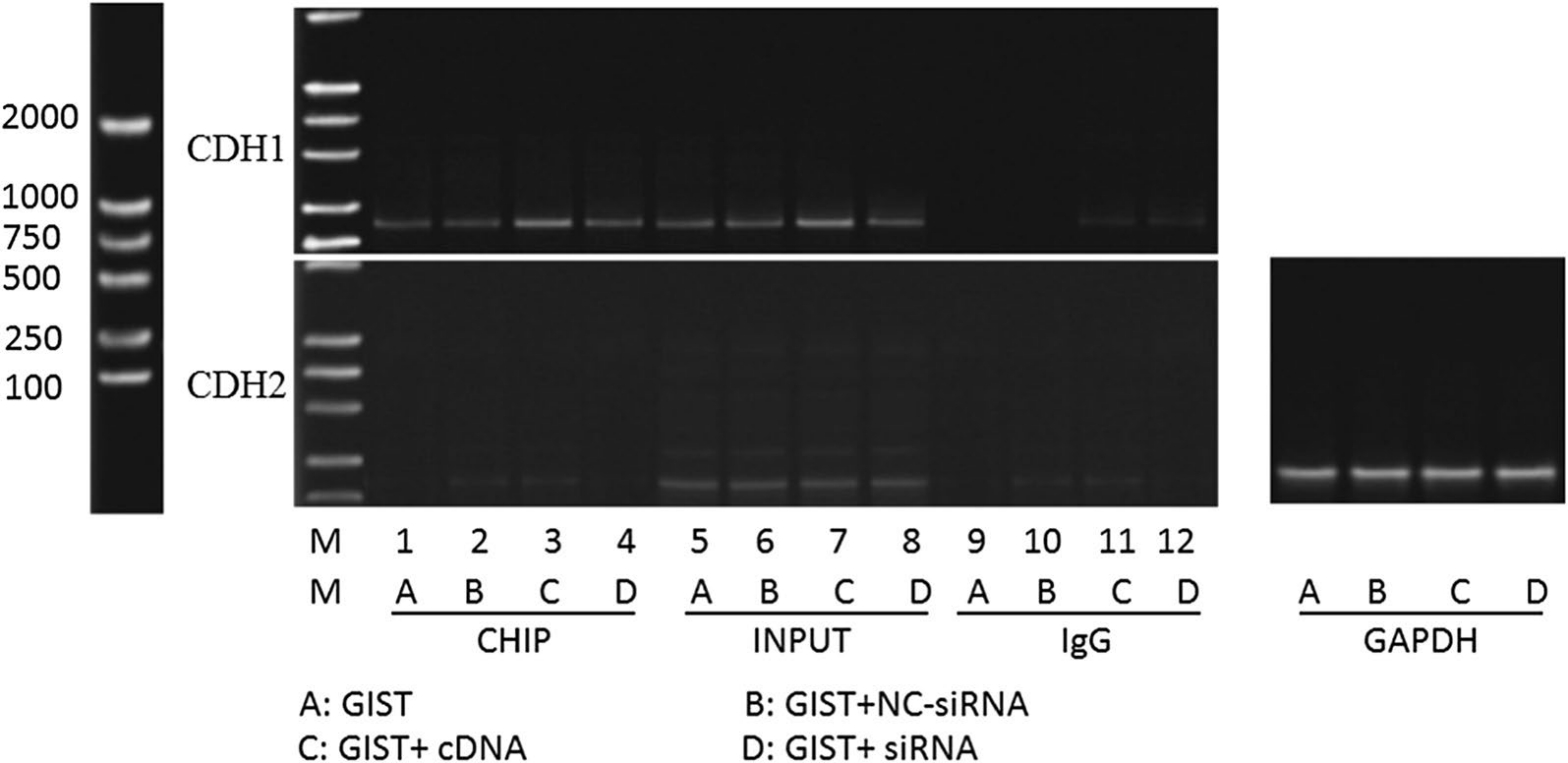
ChIP assay in GIST cells. SNAI2 was present at the proximal promoter of CDH-1, but not at the proximal promoter of CDH2, in GIST cells. Its level at the proximal promoter of CDH-1 was significantly higher in SNAI2 overexpression group and lower in SNAI2 knockdown group

Considering that knockout of SNAI2 was accompanied by the upregulation of CDH-1, the inhibition of invasive ability of GIST cells, and the invariable expression of CDH-2, we speculated that SNAI2 facilitates the metastasis of GISTs by inhibiting CDH-1 expression. ChIP analysis was performed to determine whether the promoters of CDH-1 and CDH-2 are directly regulated by SNAI2, which contributes to metastasis of GIST cells. Results confirmed that SNAI2 was present at the proximal promoter of CDH-1 but absent at that of CDH-2. Quantitative analysis revealed that the enrichment of SNAI2 at the proximal promoter of CDH-1 significantly increased in the SNAI2 overexpression group and decreased in the SNAI2 knockdown group (Fig. 10). Therefore, we conclude that SNAI2 targetedly binds to the promoter of the CDH1 gene, downregulates the expression of CDH1, and consequently contributes to the metastasis of GISTs.

## Discussion

GISTs are common mesenchymal malignancies in the digestive tract. Nearly 40% of GISTs that are localized at the time of detection give rise to metastases [1]. Although molecular targeted drugs and surgery have significantly improved the therapeutic effects of GISTs, more than 30% of patients with GISTs relapse within 5 years [5, 6]. To date, the mechanism underlying GIST metastasis remains to be elucidated.

EMT contributes to carcinoma metastasis. SNAI2 is a prominent EMT-inducing transcription factor that facilitates tumor cell invasion, metastasis, and survival [7–10]. The regulation of tumor metastasis by SNAI2 is not only limited to epithelial-derived carcinomas but also interstitial tumors [18–26]. Yang et al. [21] found that the expression of CDH1 and SNAI2 negatively correlates in leiomyosarcoma. Silencing the SNAI2 gene could significantly upregulate the expression of CDH1, downregulate the expression of vimentin and CDH2 in leiomyosarcoma cells, and significantly impair the proliferation and invasiveness of cells. Our research also found that SNAI2 expression markedly increased and CDH1 expression markedly decreased in the GIST cases with distant metastasis. Silencing of the SNAI2 gene impaired the invasiveness of GIST cells in vitro, which demonstrated that both SNAI2 and CDH1 play an important role in GIST metastasis. This process is contrary to EMT where acquisition of a mesenchymal phenotype and loss of epithelial phenotype are associated with improved tumor cell invasiveness [42, 43]. Similarly, Pulkka et al. [44] found that SNAI2 downregulation inhibits cell proliferation, induces cell death, and increases the sensitivity of GIST cells to the treatment of imatinib mesylate. High expression of SNAI2 in GISTs indicates a worse prognosis, and they speculated that SNAI2 acts as a pro-proliferative factor in GISTs.

Our research revealed that SNAI2 protein targetedly bound to the promoter of the CDH1 gene, downregulated the expression of CDH1, and increased the invasiveness of GIST cells. SNAI2-induced inhibition of CDH1 is mediated by its binding to proximal E-boxes of the CDH1 promoter [45]. This effect is dependent on LSD1-mediated histone methylation modification [46, 47]. The SNAG domain of SNAI2 functions as a molecular hook to recruit LSD1 to the promoters of its target gene, such as CDH1, decreases the monomethylation and dimethylation of histone H3 lysine4 (H3K4) at this region, downregulates gene expression, and consequently contributes to cancer metastasis [47, 48].

MicroRNA (MiRNA) is a small endogenous non-encoded RNA with a length of about 22 nucleotides that interacts with their corresponding target mRNAs to inhibit mRNA translation into proteins [49]. SNAI2 is also regulated by a variety of mRNAs. Shi et al. found that the inhibition of miR-218 contributes to the EMT and metastasis of lung cancer by targeting the SNAI2/ZEB2 signaling pathway [15]. Interestingly, restoration of the expression of miR-200 downregulates SNAI2 and other inhibitory transcription factors, and reverses EMT in pancreatic cancer cells [36]. Results showed that miR-200b-3p, miR-30c-1-3P, and miR-363-3P targetedly bound to the 3′-UTR of SNAI2, downregulated SNAI2 expression, and inhibited the invasiveness of GIST cells, which further confirmed that miRNAs could inhibit GIST invasiveness through the SNAI2/CDH1 axis. In the near future, miRNAs may become a novel drug target for cancer therapy.

The present study has some limitations. First, aside from CDH1 and CDH2, other downstream metastasis-associated target genes of SNAI2 could be detected to illustrate the real situation of SNAI2-mediated metastasis of GISTs. Second, whether proteins such as LSD1 participate in the SNAI2-mediated metastasis of GISTs remain to be confirmed. Third, only two GIST cell lines were available, although we attempted to include more cell lines in this research. The effects of SNAI2 and their upstream miRNAs on the invasiveness of GIST cells might vary between different cell lines.

## Conclusion

The results suggest that SNAI2 and CDH1 correlate with the metastasis of GISTs, and silencing of the SNAI2 gene impairs the invasiveness of GIST cells. MiR-200b-3p, miR-30c-1-3P, and miR-363-3P contribute to the metastasis of GISTs by mediating the SNAI2/CDH1 axis. In the future, we will study their effects on the proliferation and metastasis of GISTs in vivo. SNAI2 may be a potential target for the treatment of GISTs (Additional files 1, 2).

## Supplementary information

**Additional file 1.** Prediction of the upstream miRNAs of SNAI2 by Targetscan

**Additional file 2.** The microarray profiling of miRNAs in high-SNAI2-level GISTs and low-SNAI2-level GISTs

## Data Availability

All the literatures were collected by searching the PubMed and Web of Science database. The raw data and processed data of the study were available from the corresponding author upon reasonable request.

## Abbreviations

GISTs: gastrointestinal stromal tumors
MiRNA: microRNA
CDH1: cadherin1
CDH2: cadherin2
LSD1: lysine-specific demethylase 1
EMT: epithelial–mesenchymal transition
