# Supplementary material for: Identification of upstream miRNAs of SNAI2 and their influence on the metastasis of gastrointestinal stromal tumors
Source: Cancer Cell Int. 2019 Nov 12;19:289. doi: 10.1186/s12935-019-1006-8 (PMC6852720; doi:10.1186/s12935-019-1006-8)
Supplement: Supplementary file 1 — Additional file 1. Prediction of the upstream miRNAs of SNAI2 by Targetscan [file 12935_2019_1006_MOESM1_ESM.pdf]

miRNA	Position	seed matc	context++	context++	weighted	conservec	Pct
Conserved sites							
hsa-miR-203a-3p.1	351-357	7mer-1A	-0.1	86	-0.1	3.923	0.23
hsa-miR-429	369-375	7mer-m8	-0.17	93	-0.17	3.271	0.43
hsa-miR-200b-3p	369-375	7mer-m8	-0.17	93	-0.17	3.271	0.43
hsa-miR-200c-3p	369-375	7mer-m8	-0.16	92	-0.16	3.271	0.43
hsa-miR-124-3p.1	439-446	8mer	-0.48	99	-0.48	3.489	0.74
hsa-miR-181b-5p	587-593	7mer-1A	-0.26	96	-0.26	4.394	0.45
hsa-miR-181c-5p	587-593	7mer-1A	-0.26	96	-0.26	4.394	0.45
hsa-miR-181a-5p	587-593	7mer-1A	-0.26	96	-0.26	4.394	0.45
hsa-miR-181d-5p	587-593	7mer-1A	-0.25	95	-0.25	4.394	0.45
hsa-miR-4262	587-593	7mer-1A	-0.17	94	-0.17	4.394	0.45
hsa-miR-655-3p	590-597	8mer	-0.24	95	-0.24	2.671	N/A
hsa-miR-374c-5p	590-597	8mer	-0.25	95	-0.25	2.671	N/A
hsa-miR-124-3p.1	639-645	7mer-m8	-0.32	96	-0.32	4.391	0.84
hsa-miR-206	673-679	7mer-m8	-0.27	94	-0.27	4.271	0.71
hsa-miR-1-3p	673-679	7mer-m8	-0.27	94	-0.27	4.271	0.71
hsa-miR-613	673-679	7mer-m8	-0.25	93	-0.25	4.271	0.71
hsa-miR-33a-5p	816-822	7mer-1A	-0.2	97	-0.2	3.718	0.3
hsa-miR-33b-5p	816-822	7mer-1A	-0.2	97	-0.2	3.718	0.3
hsa-miR-124-3p.1	843-849	7mer-m8	-0.48	99	-0.48	3.975	0.76
Poorly conserved sites							
hsa-miR-4444	19-25	7mer-1A	-0.34	90	-0.34	0	N/A
hsa-miR-26b-3p	23-29	7mer-1A	-0.16	86	-0.16	0.031	N/A
hsa-miR-4652-3p	24-30	7mer-m8	-0.06	88	-0.06	0	N/A
hsa-miR-4778-5p	25-31	7mer-m8	-0.14	91	-0.14	0	N/A
hsa-miR-586	29-35	7mer-m8	-0.17	90	-0.17	0.016	N/A
hsa-miR-5700	30-36	7mer-m8	-0.17	94	-0.17	0.137	N/A
hsa-miR-5680	33-39	7mer-m8	-0.02	86	-0.02	0	N/A
hsa-miR-3185	36-42	7mer-1A	-0.11	87	-0.11	0	N/A
hsa-miR-6815-3p	47-53	7mer-1A	-0.09	67	-0.09	0	N/A
hsa-miR-664b-3p	52-58	7mer-m8	-0.03	83	-0.03	0.043	N/A
hsa-miR-579-3p	52-58	7mer-m8	-0.02	65	-0.02	0.043	N/A
hsa-miR-616-3p	54-60	7mer-1A	-0.14	83	-0.14	0.016	N/A
hsa-miR-5009-5p	65-72	8mer	-0.55	99	-0.55	0.239	N/A
hsa-miR-8058	65-72	8mer	-0.56	99	-0.56	0.239	N/A
hsa-miR-4529-3p	66-73	8mer	-0.54	99	-0.54	0	N/A
hsa-miR-1269a	66-72	7mer-1A	-0.3	95	-0.3	0.223	N/A
hsa-miR-1269b	66-72	7mer-1A	-0.3	95	-0.3	0.223	N/A
hsa-miR-7850-5p	67-73	7mer-m8	-0.2	98	-0.2	0	N/A
hsa-miR-4520-2-3p	67-73	7mer-1A	-0.22	85	-0.22	0	N/A
hsa-miR-455-5p	67-78	non-canor	N/A	N/A	N/A	0	N/A
hsa-miR-455-5p	67-78	non-canor	N/A	N/A	N/A	0	N/A
hsa-miR-532-5p	72-78	7mer-m8	-0.22	94	-0.22	2.795	N/A
hsa-miR-4768-3p	81-87	7mer-m8	-0.14	90	-0.14	0.031	N/A
hsa-miR-383-3p	87-93	7mer-1A	-0.16	92	-0.16	0	N/A
hsa-miR-3065-3p	87-93	7mer-1A	-0.12	85	-0.12	0	N/A
hsa-miR-6801-5p	90-96	7mer-1A	-0.23	92	-0.23	0	N/A
hsa-miR-5587-5p	91-97	7mer-1A	-0.13	81	-0.13	0	N/A
hsa-miR-92a-1-5p	94-100	7mer-1A	-0.22	80	-0.22	0.506	N/A
hsa-miR-659-3p	96-102	7mer-1A	-0.06	75	-0.06	0	N/A
hsa-miR-5002-5p	97-104	8mer	-0.38	99	-0.38	0	N/A
hsa-miR-126-5p	101-107	7mer-1A	-0.15	95	-0.15	0.326	N/A
hsa-miR-4795-3p	101-107	7mer-m8	-0.06	81	-0.06	0	N/A

hsa-miR-656-3p	102-108	7mer-m8	-0.06	89	-0.06	0.175	N/A
hsa-miR-5580-3p	105-111	7mer-1A	-0.12	92	-0.12	0	N/A
hsa-miR-1284	108-115	8mer	-0.39	98	-0.39	0.016	N/A
hsa-miR-4796-5p	110-117	8mer	-0.34	96	-0.34	0	N/A
hsa-miR-4711-3p	113-119	7mer-1A	-0.18	88	-0.18	0	N/A
hsa-miR-6867-5p	115-121	7mer-1A	-0.09	47	-0.09	0	N/A
hsa-miR-6867-5p	116-123	8mer	-0.4	95	-0.4	0	N/A
hsa-miR-6867-5p	118-125	8mer	-0.4	95	-0.4	0	N/A
hsa-miR-511-3p	121-127	7mer-1A	-0.15	86	-0.15	0.031	N/A
hsa-miR-448	125-131	7mer-m8	-0.17	90	-0.17	0.134	N/A
hsa-miR-6867-5p	130-137	8mer	-0.36	92	-0.36	0.223	N/A
hsa-miR-574-5p	130-141	non-canor	N/A	N/A	N/A	0	N/A
hsa-miR-574-5p	130-141	non-canor	N/A	N/A	N/A	0	N/A
hsa-miR-574-5p	131-143	non-canor	N/A	N/A	N/A	0	N/A
hsa-miR-574-5p	131-143	non-canor	N/A	N/A	N/A	0	N/A
hsa-miR-6867-5p	132-139	8mer	-0.36	93	-0.36	0.223	N/A
hsa-miR-6867-5p	134-141	8mer	-0.36	92	-0.36	0.223	N/A
hsa-miR-6867-5p	136-143	8mer	-0.35	92	-0.35	0.223	N/A
hsa-miR-6867-5p	138-145	8mer	-0.35	92	-0.35	0.223	N/A
hsa-miR-6867-5p	140-147	8mer	-0.34	91	-0.34	0.223	N/A
hsa-miR-6867-5p	142-148	7mer-m8	-0.18	70	-0.18	0.223	N/A
hsa-miR-3650	143-149	7mer-m8	-0.25	93	-0.25	0	N/A
hsa-miR-4455	144-151	8mer	-0.55	98	-0.55	0	N/A
hsa-miR-6772-5p	145-151	7mer-1A	-0.22	87	-0.22	0	N/A
hsa-miR-609	145-151	7mer-1A	-0.15	77	-0.15	0	N/A
hsa-miR-6748-5p	146-153	8mer	-0.48	98	-0.48	0	N/A
hsa-miR-6759-5p	147-153	7mer-m8	-0.24	93	-0.24	0	N/A
hsa-miR-6793-5p	147-153	7mer-1A	-0.16	82	-0.16	0	N/A
hsa-miR-134-3p	148-155	8mer	-0.47	99	-0.47	0	N/A
hsa-miR-7114-5p	149-155	7mer-1A	-0.19	91	-0.19	0	N/A
hsa-miR-6817-3p	152-159	8mer	-0.33	98	-0.33	0	N/A
hsa-miR-6873-3p	153-159	7mer-m8	-0.13	80	-0.13	0	N/A
hsa-miR-7110-3p	153-159	7mer-1A	-0.04	56	-0.04	0	N/A
hsa-miR-7110-3p	154-161	8mer	-0.37	96	-0.37	0	N/A
hsa-miR-6817-3p	155-161	7mer-1A	-0.1	79	-0.1	0	N/A
hsa-miR-6873-3p	155-161	7mer-m8	-0.12	78	-0.12	0	N/A
hsa-miR-7110-3p	156-162	7mer-m8	-0.23	89	-0.23	0	N/A
hsa-miR-4660	160-167	8mer	-0.49	99	-0.49	0.016	N/A
hsa-miR-1301-3p	161-168	8mer	-0.41	98	-0.41	0.137	N/A
hsa-miR-5047	161-168	8mer	-0.43	98	-0.43	0.137	N/A
hsa-miR-7156-3p	162-168	7mer-1A	-0.15	82	-0.15	0	N/A
hsa-miR-5581-3p	171-178	8mer	-0.44	99	-0.44	0	N/A
hsa-miR-6872-3p	171-177	7mer-1A	-0.15	80	-0.15	0	N/A
hsa-miR-4452	176-182	7mer-m8	-0.12	91	-0.12	0	N/A
hsa-miR-4643	181-187	7mer-m8	-0.15	96	-0.15	0	N/A
hsa-miR-302b-5p	188-194	7mer-1A	-0.04	85	-0.04	0	N/A
hsa-miR-302d-5p	188-194	7mer-1A	-0.01	72	-0.01	0	N/A
hsa-miR-3159	195-201	7mer-m8	-0.22	95	-0.22	0	N/A
hsa-miR-541-5p	196-202	7mer-m8	-0.21	95	-0.21	0.175	N/A
hsa-miR-3978	200-206	7mer-m8	-0.14	96	-0.14	0	N/A
hsa-miR-3688-3p	201-207	7mer-m8	-0.09	82	-0.09	0	N/A
hsa-miR-4802-3p	203-209	7mer-m8	-0.24	95	-0.24	0	N/A
hsa-miR-4717-3p	204-211	8mer	-0.49	99	-0.49	0	N/A
hsa-miR-23b-3p	206-212	7mer-1A	-0.14	85	-0.14	2.684	0.11

hsa-miR-23a-3p	206-212	7mer-1A	-0.13	83	-0.13	2.684	0.11
hsa-miR-23c	206-212	7mer-1A	-0.11	80	-0.11	2.684	0.11
hsa-miR-130a-5p	206-212	7mer-1A	-0.04	62	-0.04	2.684	0.11
hsa-miR-590-3p	216-223	8mer	-0.03	79	-0.03	0.327	N/A
hsa-miR-4775	217-223	7mer-m8	-0.04	90	-0.04	0	N/A
hsa-miR-5011-5p	225-231	7mer-m8	-0.05	82	-0.05	0	N/A
hsa-miR-1277-5p	226-232	7mer-m8	-0.05	85	-0.05	0	N/A
hsa-miR-545-3p	231-238	8mer	-0.38	99	-0.38	0.043	N/A
hsa-miR-3617-3p	233-239	7mer-m8	-0.27	93	-0.27	0.031	N/A
hsa-miR-1287-3p	240-246	7mer-m8	-0.39	96	-0.39	0	N/A
hsa-miR-6833-3p	249-255	7mer-m8	-0.26	96	-0.26	0	N/A
hsa-miR-4768-5p	249-255	7mer-m8	-0.28	96	-0.28	0	N/A
hsa-miR-6873-3p	249-255	7mer-1A	-0.16	85	-0.16	0	N/A
hsa-miR-511-5p	258-264	7mer-m8	-0.11	92	-0.11	0.016	N/A
hsa-miR-6830-3p	258-264	7mer-1A	-0.15	90	-0.15	0	N/A
hsa-miR-4693-5p	262-268	7mer-1A	-0.17	85	-0.17	0	N/A
hsa-miR-6730-5p	268-274	7mer-m8	-0.22	96	-0.22	0	N/A
hsa-miR-4709-3p	274-281	8mer	-0.39	99	-0.39	0.137	N/A
hsa-miR-153-5p	286-292	7mer-1A	-0.02	49	-0.02	0	N/A
hsa-miR-1250-3p	286-292	7mer-1A	-0.01	46	-0.01	0	N/A
hsa-miR-664b-3p	287-293	7mer-1A	-0.07	91	-0.07	0.016	N/A
hsa-miR-5696	287-293	7mer-1A	-0.1	90	-0.1	0	N/A
hsa-miR-579-3p	287-293	7mer-1A	-0.07	90	-0.07	0.016	N/A
hsa-miR-335-3p	289-295	7mer-m8	-0.08	92	-0.08	0.031	N/A
hsa-miR-3140-3p	292-298	7mer-1A	-0.13	87	-0.13	0	N/A
hsa-miR-218-5p	294-300	7mer-m8	-0.3	95	-0.3	2.367	0.19
hsa-miR-636	294-300	7mer-1A	-0.17	88	-0.17	0.031	N/A
hsa-miR-106a-3p	299-305	7mer-m8	-0.21	95	-0.21	0.223	N/A
hsa-miR-6876-5p	312-318	7mer-1A	-0.18	92	-0.18	0	N/A
hsa-miR-4476	312-318	7mer-1A	-0.17	91	-0.17	0	N/A
hsa-miR-651-5p	314-320	7mer-1A	-0.14	88	-0.14	0.016	N/A
hsa-miR-153-5p	320-326	7mer-m8	-0.02	50	-0.02	0	N/A
hsa-miR-33a-5p	323-329	7mer-1A	-0.11	91	-0.11	2.337	< 0.1
hsa-miR-33b-5p	323-329	7mer-1A	-0.11	91	-0.11	2.337	< 0.1
hsa-miR-3680-3p	324-330	7mer-m8	-0.18	94	-0.18	0	N/A
hsa-miR-4495	333-339	7mer-m8	-0.11	89	-0.11	0	N/A
hsa-miR-3908	337-343	7mer-m8	-0.17	96	-0.17	0	N/A
hsa-miR-885-3p	341-347	7mer-1A	-0.33	98	-0.33	0.043	N/A
hsa-miR-7150	342-348	7mer-1A	-0.18	84	-0.18	0	N/A
hsa-miR-182-5p	343-349	7mer-1A	-0.23	89	-0.23	3.345	0.4
hsa-miR-1271-5p	343-349	7mer-1A	-0.16	84	-0.16	3.345	0.4
hsa-miR-96-5p	343-349	7mer-1A	-0.13	81	-0.13	3.345	0.4
hsa-miR-580-5p	348-354	7mer-m8	-0.11	92	-0.11	0	N/A
hsa-miR-30d-3p	358-365	8mer	-0.29	98	-0.29	0.164	N/A
hsa-miR-30e-3p	358-365	8mer	-0.28	98	-0.28	0.164	N/A
hsa-miR-30a-3p	358-365	8mer	-0.29	98	-0.29	0.164	N/A
hsa-miR-627-3p	361-367	7mer-1A	-0.04	66	-0.04	0	N/A
hsa-miR-4677-5p	364-370	7mer-m8	-0.15	90	-0.15	0.031	N/A
hsa-miR-2116-5p	364-370	7mer-1A	-0.11	89	-0.11	0	N/A
hsa-miR-22-5p	364-370	7mer-1A	-0.08	72	-0.08	0.159	N/A
hsa-miR-26b-3p	365-371	7mer-m8	-0.23	92	-0.23	0.031	N/A
hsa-miR-892c-3p	367-373	7mer-1A	-0.11	84	-0.11	2.448	N/A
hsa-miR-452-5p	367-373	7mer-1A	-0.11	83	-0.11	2.448	N/A
hsa-miR-4676-3p	367-373	7mer-1A	-0.09	80	-0.09	2.448	N/A

hsa-miR-3942-5p	372-378	7mer-m8	-0.17	91	-0.17	0	N/A
hsa-miR-4703-5p	372-378	7mer-m8	-0.14	89	-0.14	0	N/A
hsa-miR-330-3p	375-381	7mer-m8	-0.16	95	-0.16	1.526	N/A
hsa-miR-524-5p	377-384	8mer	-0.18	98	-0.18	0.031	N/A
hsa-miR-520d-5p	377-384	8mer	-0.17	98	-0.17	0.031	N/A
hsa-miR-6504-3p	380-386	7mer-1A	-0.06	89	-0.06	0	N/A
hsa-miR-6504-3p	391-398	8mer	-0.16	97	-0.16	0	N/A
hsa-miR-3153	402-408	7mer-1A	-0.08	84	-0.08	0	N/A
hsa-miR-6733-5p	402-408	7mer-1A	-0.12	83	-0.12	0	N/A
hsa-miR-4668-5p	402-408	7mer-1A	-0.12	80	-0.12	0	N/A
hsa-miR-6739-5p	402-408	7mer-1A	-0.11	80	-0.11	0	N/A
hsa-miR-6871-5p	404-410	7mer-1A	-0.14	77	-0.14	0	N/A
hsa-miR-202-5p	407-414	8mer	-0.25	96	-0.25	0.314	< 0.1
hsa-miR-337-3p	408-414	7mer-1A	-0.13	77	-0.13	0.043	N/A
hsa-miR-1236-3p	411-418	8mer	-0.33	97	-0.33	0.016	N/A
hsa-miR-6515-3p	412-418	7mer-1A	-0.1	78	-0.1	0	N/A
hsa-miR-6868-5p	420-427	8mer	-0.43	99	-0.43	0	N/A
hsa-miR-3128	421-427	7mer-1A	-0.16	87	-0.16	0.223	N/A
hsa-miR-4720-5p	421-427	7mer-1A	-0.11	83	-0.11	0	N/A
hsa-miR-4799-3p	421-427	7mer-1A	-0.12	82	-0.12	0	N/A
hsa-miR-5588-5p	421-427	7mer-1A	-0.09	77	-0.09	0	N/A
hsa-miR-4682	432-438	7mer-m8	-0.06	71	-0.06	0	N/A
hsa-miR-3622b-3p	435-441	7mer-m8	-0.21	96	-0.21	0	N/A
hsa-miR-3622a-3p	435-441	7mer-m8	-0.18	93	-0.18	0	N/A
hsa-miR-6804-3p	436-442	7mer-m8	-0.28	93	-0.28	0	N/A
hsa-miR-506-3p	440-447	8mer	-0.32	95	-0.32	1.18	< 0.1
hsa-miR-124-3p.2	440-447	8mer	-0.36	94	-0.36	1.18	< 0.1
hsa-miR-3910	440-446	7mer-1A	-0.1	84	-0.1	0	N/A
hsa-miR-3714	440-446	7mer-1A	-0.07	83	-0.07	0	N/A
hsa-miR-7856-5p	442-449	8mer	-0.16	97	-0.16	0	N/A
hsa-miR-4477b	442-448	7mer-1A	-0.14	82	-0.14	0	N/A
hsa-miR-5003-3p	447-453	7mer-1A	-0.01	66	-0.01	0	N/A
hsa-miR-6505-5p	452-459	8mer	-0.26	97	-0.26	0.031	N/A
hsa-miR-890	455-461	7mer-m8	-0.25	96	-0.25	0.333	N/A
hsa-miR-6514-5p	464-470	7mer-m8	-0.32	98	-0.32	0	N/A
hsa-miR-380-3p	469-475	7mer-m8	-0.05	76	-0.05	0.244	N/A
hsa-miR-196a-3p	488-494	7mer-1A	-0.22	87	-0.22	0	N/A
hsa-miR-5197-5p	490-496	7mer-m8	-0.16	83	-0.16	0	N/A
hsa-miR-7159-5p	495-502	8mer	-0.22	96	-0.22	0	N/A
hsa-miR-3065-5p	495-501	7mer-1A	-0.12	85	-0.12	0	N/A
hsa-miR-421	496-502	7mer-1A	-0.13	84	-0.13	1.352	N/A
hsa-miR-505-3p.2	496-502	7mer-1A	-0.04	83	-0.04	1.352	N/A
hsa-miR-5000-5p	499-505	7mer-1A	-0.15	89	-0.15	0	N/A
hsa-miR-3613-3p	512-519	8mer	-0.07	84	-0.07	0	N/A
hsa-miR-552-3p	522-528	7mer-1A	-0.2	88	-0.2	0.016	N/A
hsa-miR-6835-3p	529-536	8mer	-0.37	99	-0.37	0.134	N/A
hsa-miR-4422	530-536	7mer-1A	-0.09	90	-0.09	0	N/A
hsa-miR-9-3p	531-537	7mer-1A	-0.13	85	-0.13	0.666	N/A
hsa-miR-548c-3p	543-549	7mer-1A	-0.01	57	-0.01	0.016	N/A
hsa-miR-6832-3p	549-556	8mer	-0.37	98	-0.37	0	N/A
hsa-miR-204-5p	550-556	7mer-m8	-0.21	93	-0.21	0.091	< 0.1
hsa-miR-211-5p	550-556	7mer-m8	-0.2	92	-0.2	0.091	< 0.1
hsa-miR-4287	551-557	7mer-m8	-0.29	97	-0.28	0	N/A
hsa-miR-4685-3p	551-557	7mer-m8	-0.25	96	-0.25	0	N/A

hsa-miR-6749-3p	553-560	8mer	-0.57	99	-0.57	0	N/A
hsa-miR-4691-5p	554-560	7mer-1A	-0.16	91	-0.16	0	N/A
hsa-miR-6792-3p	554-560	7mer-1A	-0.21	90	-0.21	0	N/A
hsa-miR-1238-3p	555-561	7mer-1A	-0.15	92	-0.15	0	N/A
hsa-miR-670-3p	555-561	7mer-1A	-0.15	87	-0.15	0.487	N/A
hsa-miR-578	568-574	7mer-m8	-0.15	92	-0.15	0.239	N/A
hsa-miR-4677-5p	570-577	8mer	-0.31	98	-0.31	0.031	N/A
hsa-miR-2116-5p	570-576	7mer-1A	-0.08	83	-0.08	0	N/A
hsa-miR-22-5p	570-576	7mer-1A	-0.04	57	-0.04	0.26	N/A
hsa-miR-6739-3p	571-578	8mer	-0.38	98	-0.37	0	N/A
hsa-miR-4273	571-577	7mer-1A	-0.04	80	-0.04	0	N/A
hsa-miR-7156-5p	571-577	7mer-1A	-0.07	78	-0.07	0	N/A
hsa-miR-375	572-578	7mer-1A	-0.17	96	-0.17	1.264	< 0.1
hsa-miR-19a-5p	575-581	7mer-1A	-0.1	85	-0.1	0.031	N/A
hsa-miR-19b-2-5p	575-581	7mer-1A	-0.1	84	-0.1	0.031	N/A
hsa-miR-19b-1-5p	575-581	7mer-1A	-0.1	84	-0.1	0.031	N/A
hsa-miR-2052	575-581	7mer-1A	-0.12	83	-0.11	0	N/A
hsa-miR-1914-5p	580-586	7mer-1A	-0.15	87	-0.15	0.137	N/A
hsa-miR-3653-5p	582-588	7mer-1A	-0.26	98	-0.26	0	N/A
hsa-miR-1976	582-588	7mer-1A	-0.16	90	-0.16	0	N/A
hsa-miR-4279	583-589	7mer-1A	-0.12	86	-0.12	0	N/A
hsa-miR-5003-3p	596-602	7mer-1A	-0.05	87	-0.05	0	N/A
hsa-miR-4470	621-627	7mer-1A	-0.09	83	-0.09	0	N/A
hsa-miR-182-5p	622-628	7mer-m8	-0.29	94	-0.29	1.344	0.12
hsa-miR-569	628-634	7mer-1A	-0.09	75	-0.09	0.043	N/A
hsa-miR-2053	628-634	7mer-m8	-0.02	67	-0.02	0	N/A
hsa-miR-452-5p	631-637	7mer-1A	-0.11	84	-0.11	0.971	N/A
hsa-miR-4676-3p	631-637	7mer-1A	-0.1	81	-0.09	0.971	N/A
hsa-miR-892c-3p	631-637	7mer-1A	-0.08	77	-0.08	0.971	N/A
hsa-miR-5582-5p	638-644	7mer-m8	-0.24	93	-0.24	0	N/A
hsa-miR-6814-5p	642-648	7mer-m8	-0.15	90	-0.15	0	N/A
hsa-miR-361-3p	645-651	7mer-1A	-0.14	79	-0.14	0.043	N/A
hsa-miR-6749-3p	648-654	7mer-m8	-0.13	72	-0.13	0	N/A
hsa-miR-5193	650-656	7mer-m8	-0.12	77	-0.12	0.137	N/A
hsa-miR-4667-3p	651-658	8mer	-0.27	95	-0.27	0	N/A
hsa-miR-1470	652-658	7mer-1A	-0.13	91	-0.13	0	N/A
hsa-miR-642a-5p	653-659	7mer-1A	-0.01	34	-0.01	0.043	N/A
hsa-miR-4446-5p	654-660	7mer-m8	-0.03	59	-0.03	0	N/A
hsa-miR-5006-3p	654-660	7mer-1A	-0.03	57	-0.03	0	N/A
hsa-miR-4755-5p	654-660	7mer-1A	-0.01	43	-0.01	0	N/A
hsa-miR-3124-3p	656-662	7mer-1A	-0.15	90	-0.15	0.223	N/A
hsa-miR-330-3p	666-672	7mer-1A	-0.13	94	-0.13	1.522	N/A
hsa-miR-4787-3p	681-687	7mer-m8	-0.34	92	-0.34	0	N/A
hsa-miR-6081	687-693	7mer-1A	-0.32	95	-0.32	0	N/A
hsa-miR-3617-5p	695-701	7mer-1A	-0.16	95	-0.16	0.016	N/A
hsa-miR-641	695-701	7mer-1A	-0.14	93	-0.13	0.016	N/A
hsa-miR-4307	709-715	7mer-m8	-0.16	96	-0.16	0	N/A
hsa-miR-33a-3p	710-716	7mer-m8	-0.16	96	-0.16	0	N/A
hsa-miR-3662	713-719	7mer-1A	-0.08	98	-0.08	0	N/A
hsa-miR-4477a	717-723	7mer-1A	-0.09	90	-0.09	0	N/A
hsa-miR-126-5p	718-724	7mer-m8	-0.09	89	-0.09	0	N/A
hsa-miR-944	719-725	7mer-m8	-0.1	95	-0.1	0.016	N/A
hsa-miR-3613-3p	724-730	7mer-1A	-0.09	89	-0.09	0	N/A
hsa-miR-607	726-732	7mer-1A	-0.01	40	-0.01	0	N/A

hsa-miR-1305	727-733	7mer-1A	-0.02	78	-0.02	0	N/A
hsa-miR-4775	730-737	8mer	-0.03	79	-0.03	0	N/A
hsa-miR-590-3p	730-736	7mer-1A	-0.01	34	-0.01	0.164	N/A
hsa-miR-302d-5p	738-744	7mer-1A	-0.02	80	-0.02	0.137	N/A
hsa-miR-302b-5p	738-744	7mer-1A	-0.02	78	-0.02	0.137	N/A
hsa-miR-3938	746-752	7mer-1A	-0.23	92	-0.23	0.031	N/A
hsa-miR-4325	752-759	8mer	-0.47	99	-0.47	0	N/A
hsa-miR-7703	753-759	7mer-1A	-0.14	88	-0.13	0	N/A
hsa-miR-557	754-761	8mer	-0.42	99	-0.42	0.043	N/A
hsa-miR-507	754-761	8mer	-0.48	99	-0.48	0.043	N/A
hsa-miR-450b-5p	755-761	7mer-1A	-0.16	93	-0.16	0.031	N/A
hsa-miR-3680-3p	755-761	7mer-1A	-0.07	82	-0.07	0	N/A
hsa-miR-5187-3p	758-770	non-canor	N/A	N/A	N/A	0	N/A
hsa-miR-5187-3p	758-770	non-canor	N/A	N/A	N/A	0	N/A
hsa-miR-6881-3p	760-766	7mer-m8	-0.3	96	-0.3	0	N/A
hsa-miR-877-3p	760-766	7mer-1A	-0.2	89	-0.2	0	N/A
hsa-miR-6507-5p	766-772	7mer-1A	-0.03	80	-0.03	0	N/A
hsa-miR-3618	778-789	non-canor	N/A	N/A	N/A	0	N/A
hsa-miR-3618	778-789	non-canor	N/A	N/A	N/A	0	N/A
hsa-miR-323a-3p	782-788	7mer-1A	-0.03	80	-0.03	1.76	N/A
hsa-miR-510-3p	798-804	7mer-1A	-0.07	92	-0.07	0	N/A
hsa-miR-586	803-810	8mer	-0.38	98	-0.38	0.016	N/A
hsa-miR-4536-5p	809-815	7mer-1A	-0.22	83	-0.21	0.043	N/A
hsa-miR-25-3p	818-824	7mer-1A	-0.16	89	-0.16	3.362	0.22
hsa-miR-363-3p	818-824	7mer-1A	-0.16	88	-0.16	3.362	0.22
hsa-miR-367-3p	818-824	7mer-1A	-0.16	88	-0.16	3.362	0.22
hsa-miR-92a-3p	818-824	7mer-1A	-0.14	87	-0.14	3.362	0.22
hsa-miR-32-5p	818-824	7mer-1A	-0.14	86	-0.14	3.362	0.22
hsa-miR-92b-3p	818-824	7mer-1A	-0.11	85	-0.11	3.362	0.22
hsa-miR-137	819-825	7mer-1A	-0.12	68	-0.12	1.122	< 0.1
hsa-miR-126-5p	821-827	7mer-1A	-0.06	85	-0.06	0	N/A
hsa-miR-4795-3p	821-827	7mer-m8	-0.02	43	-0.02	0	N/A
hsa-miR-496.2	823-829	7mer-1A	-0.11	83	-0.1	0.934	N/A
hsa-miR-5681b	828-834	7mer-m8	-0.25	95	-0.25	0	N/A
hsa-miR-503-3p	829-835	7mer-m8	-0.2	89	-0.2	0	N/A
hsa-miR-3936	832-838	7mer-m8	-0.32	97	-0.32	0	N/A
hsa-miR-3179	833-839	7mer-m8	-0.33	96	-0.33	0	N/A
hsa-miR-3202	834-841	8mer	-0.42	99	-0.42	0	N/A
hsa-miR-4533	835-841	7mer-1A	-0.15	86	-0.15	0	N/A
hsa-miR-6079	836-842	7mer-1A	-0.03	66	-0.03	0	N/A
hsa-miR-890	838-844	7mer-m8	-0.22	94	-0.22	0.333	N/A
hsa-miR-3614-5p	839-845	7mer-m8	-0.26	96	-0.26	0	N/A
hsa-miR-548an	844-850	7mer-m8	-0.25	96	-0.25	0	N/A
hsa-miR-7849-3p	854-860	7mer-1A	-0.09	95	-0.09	0	N/A
hsa-miR-4666a-3p	855-862	8mer	-0.23	98	-0.22	0	N/A
hsa-miR-381-3p	856-862	7mer-1A	-0.09	92	-0.09	1.676	N/A
hsa-miR-300	856-862	7mer-1A	-0.06	88	-0.06	1.676	N/A
hsa-let-7b-3p	856-862	7mer-m8	-0.09	84	-0.09	0.031	N/A
hsa-miR-98-3p	856-862	7mer-m8	-0.09	83	-0.09	0.031	N/A
hsa-let-7f-1-3p	856-862	7mer-m8	-0.09	82	-0.09	0.031	N/A
hsa-let-7a-3p	856-862	7mer-m8	-0.08	80	-0.08	0.031	N/A
hsa-miR-1284	857-863	7mer-m8	-0.26	93	-0.26	0.016	N/A
hsa-miR-4719	881-887	7mer-m8	-0.16	96	-0.16	0	N/A
hsa-miR-548c-3p	891-897	7mer-1A	-0.01	57	-0.01	0.17	N/A

hsa-miR-3163	893-899	7mer-1A	-0.01	45	-0.01	0	N/A
hsa-miR-4705	900-906	7mer-1A	-0.12	84	-0.11	0	N/A
hsa-miR-6758-3p	905-911	7mer-m8	-0.23	96	-0.23	0	N/A
hsa-miR-485-3p	915-922	8mer	-0.27	97	-0.27	0.25	N/A
hsa-miR-539-3p	915-922	8mer	-0.34	96	-0.34	0.25	N/A
hsa-miR-4328	921-927	7mer-1A	-0.05	78	-0.05	0	N/A
hsa-miR-4797-3p	923-929	7mer-1A	-0.22	95	-0.22	0	N/A
hsa-miR-4264	923-929	7mer-1A	-0.17	90	-0.17	0	N/A
hsa-miR-1264	962-968	7mer-m8	-0.12	89	-0.12	0.016	N/A
hsa-miR-6882-5p	964-970	7mer-m8	-0.25	95	-0.25	0	N/A
hsa-miR-125b-2-3p	965-972	8mer	-0.41	99	-0.41	0	N/A
hsa-miR-513b-5p	966-973	8mer	-0.39	99	-0.39	0.043	N/A
hsa-miR-4457	966-972	7mer-1A	-0.15	88	-0.15	0	N/A
hsa-miR-494-3p	978-984	7mer-m8	-0.2	97	-0.2	1.332	N/A
hsa-miR-5582-3p	1005-101	7mer-m8	-0.17	98	-0.17	0	N/A
hsa-miR-548x-3p	1006-101	7mer-m8	-0.17	99	-0.16	0	N/A
hsa-miR-548aj-3p	1006-101	7mer-m8	-0.17	99	-0.16	0	N/A
hsa-miR-548aq-3p	1006-101	7mer-m8	-0.15	98	-0.15	0	N/A
hsa-miR-548ae-3p	1006-101	7mer-m8	-0.15	98	-0.15	0	N/A
hsa-miR-548j-3p	1006-101	7mer-m8	-0.14	98	-0.14	0	N/A
hsa-miR-548ah-3p	1006-101	7mer-m8	-0.13	98	-0.13	0	N/A
hsa-miR-548am-3p	1006-101	7mer-m8	-0.13	98	-0.13	0	N/A
hsa-miR-5701	1016-102	7mer-1A	-0.1	85	-0.1	0	N/A
hsa-miR-452-5p	1021-102	7mer-m8	-0.29	97	-0.29	0.134	N/A
hsa-miR-892c-3p	1021-102	7mer-m8	-0.26	96	-0.26	0.134	N/A
hsa-miR-4676-3p	1021-102	7mer-m8	-0.25	96	-0.25	0.134	N/A
hsa-miR-548g-3p	1023-102	7mer-m8	-0.26	98	-0.26	0	N/A
hsa-miR-548ar-3p	1024-103	7mer-m8	-0.25	99	-0.25	0.016	N/A
hsa-miR-548a-3p	1024-103	7mer-m8	-0.2	98	-0.19	0.016	N/A
hsa-miR-548f-3p	1024-103	7mer-m8	-0.2	98	-0.2	0.016	N/A
hsa-miR-548e-3p	1024-103	7mer-m8	-0.19	98	-0.19	0.016	N/A
hsa-miR-548az-3p	1024-103	7mer-m8	-0.17	98	-0.17	0.016	N/A
hsa-miR-1323	1025-103	8mer	-0.38	99	-0.38	0.043	N/A
hsa-miR-548o-3p	1025-103	8mer	-0.43	99	-0.43	0.043	N/A
hsa-miR-551b-5p	1029-103	8mer	-0.28	99	-0.28	0.031	N/A
hsa-miR-300	1046-105	7mer-1A	-0.07	90	0	0.795	N/A
hsa-miR-381-3p	1046-105	7mer-1A	-0.06	87	0	0.795	N/A
hsa-miR-4666a-3p	1046-105	7mer-1A	-0.06	86	0	0	N/A
hsa-let-7f-1-3p	1046-105	7mer-m8	-0.08	79	0	0.031	N/A
hsa-let-7a-3p	1046-105	7mer-m8	-0.05	72	0	0.031	N/A
hsa-miR-98-3p	1046-105	7mer-m8	-0.05	71	0	0.031	N/A
hsa-let-7b-3p	1046-105	7mer-m8	-0.04	68	0	0.031	N/A
hsa-miR-4766-5p	1057-106	7mer-1A	-0.16	96	0	0.031	N/A
hsa-miR-146a-3p	1058-106	8mer	-0.42	99	0	0	N/A
hsa-miR-3921	1059-106	7mer-1A	-0.18	93	0	0	N/A
hsa-miR-301b-5p	1059-106	7mer-1A	-0.08	89	0	0.031	N/A
hsa-miR-301a-5p	1059-106	7mer-1A	-0.08	89	0	0.031	N/A
hsa-miR-4653-5p	1059-106	7mer-1A	-0.15	89	0	0	N/A
hsa-miR-593-3p	1060-106	7mer-m8	-0.33	97	0	0.043	N/A
hsa-miR-3184-3p	1062-106	7mer-m8	-0.27	97	0	0	N/A
hsa-miR-6507-3p	1064-107	7mer-1A	-0.09	83	0	0	N/A
hsa-miR-8060	1075-108	8mer	-0.36	99	0	0	N/A
hsa-miR-7154-5p	1076-108	7mer-m8	-0.2	93	0	0	N/A
hsa-miR-150-3p	1086-109	7mer-1A	-0.26	89	0	0.031	N/A

hsa-miR-1263	1086-1092	7mer-1A	-0.23	86	0	0.016	N/A
hsa-miR-5087	1090-1096	7mer-1A	-0.05	68	0	0	N/A
hsa-miR-140-5p	1092-1098	7mer-m8	-0.25	93	0	0.093	< 0.1
hsa-let-7g-3p	1097-1104	8mer	-0.44	99	0	0.031	N/A
hsa-let-7a-2-3p	1097-1104	8mer	-0.43	99	0	0.031	N/A
hsa-let-7c-3p	1098-1104	7mer-1A	-0.13	86	0	0.031	N/A
hsa-miR-493-5p	1098-1104	7mer-1A	-0.1	83	0	0.093	N/A
hsa-miR-6844	1101-1108	8mer	-0.33	98	0	0	N/A
hsa-miR-495-3p	1111-1118	8mer	-0.25	99	0	0.222	N/A
hsa-miR-5688	1111-1118	8mer	-0.24	99	0	0.222	N/A
hsa-miR-7-1-3p	1112-1118	7mer-1A	-0.04	92	0	0	N/A
hsa-miR-7-2-3p	1112-1118	7mer-1A	-0.04	92	0	0	N/A
hsa-miR-2053	1116-1122	7mer-1A	-0.02	65	0	0	N/A
hsa-miR-578	1119-1126	8mer	-0.37	99	0	0	N/A
hsa-miR-153-5p	1126-1133	8mer	-0.19	98	0	0	N/A
hsa-miR-1250-3p	1127-1133	7mer-1A	-0.02	66	0	0	N/A
hsa-miR-579-3p	1128-1134	7mer-1A	-0.1	94	0	0.016	N/A
hsa-miR-5696	1128-1134	7mer-1A	-0.13	93	0	0	N/A
hsa-miR-664b-3p	1128-1134	7mer-1A	-0.09	93	0	0.016	N/A
hsa-miR-664a-3p	1129-1136	8mer	-0.29	99	0	0.016	N/A
hsa-miR-1245b-3p	1149-1155	7mer-1A	-0.2	93	0	0	N/A
hsa-miR-3942-3p	1150-1156	7mer-m8	-0.12	90	0	0	N/A
hsa-miR-5100	1150-1156	7mer-1A	-0.05	75	0	0	N/A
hsa-miR-200a-3p	1156-1162	7mer-1A	-0.13	86	0	0.892	< 0.1
hsa-miR-141-3p	1156-1162	7mer-1A	-0.13	85	0	0.892	< 0.1
hsa-miR-3163	1162-1168	7mer-m8	-0.02	65	0	0	N/A
hsa-miR-4789-3p	1166-1172	7mer-m8	-0.14	91	0	0	N/A
hsa-miR-374a-5p	1176-1182	7mer-1A	-0.12	94	0	0.719	N/A
hsa-miR-374b-5p	1176-1182	7mer-1A	-0.12	93	0	0.719	N/A
hsa-miR-4795-3p	1179-1185	7mer-1A	-0.09	86	0	0	N/A
hsa-miR-646	1190-1197	8mer	-0.62	99	0	0.016	N/A
hsa-miR-503-5p	1191-1197	7mer-1A	-0.25	97	0	0.093	< 0.1
hsa-miR-4524a-5p	1191-1197	7mer-m8	-0.37	96	0	0	N/A
hsa-miR-4524b-5p	1191-1197	7mer-m8	-0.36	96	0	0	N/A
hsa-miR-424-5p	1191-1197	7mer-1A	-0.28	94	0	0.093	< 0.1
hsa-miR-16-5p	1191-1197	7mer-1A	-0.28	94	0	0.093	< 0.1
hsa-miR-497-5p	1191-1197	7mer-1A	-0.27	94	0	0.093	< 0.1
hsa-miR-195-5p	1191-1197	7mer-1A	-0.28	94	0	0.093	< 0.1
hsa-miR-15b-5p	1191-1197	7mer-1A	-0.26	93	0	0.093	< 0.1
hsa-miR-15a-5p	1191-1197	7mer-1A	-0.26	93	0	0.093	< 0.1
hsa-miR-6838-5p	1191-1197	7mer-1A	-0.24	91	0	0.093	< 0.1
hsa-miR-4661-5p	1194-1200	7mer-1A	-0.26	90	0	0	N/A
hsa-miR-8084	1196-1203	8mer	-0.18	98	0	0	N/A
hsa-miR-429	1197-1203	7mer-1A	-0.13	88	0	0.131	< 0.1
hsa-miR-200b-3p	1197-1203	7mer-1A	-0.13	88	0	0.131	< 0.1
hsa-miR-200c-3p	1197-1203	7mer-1A	-0.13	88	0	0.131	< 0.1
hsa-miR-369-3p	1198-1204	7mer-m8	-0.22	98	0	0.094	N/A
hsa-miR-5692a	1200-1206	7mer-m8	-0.09	98	0	0	N/A
hsa-miR-548c-3p	1203-1209	7mer-1A	-0.01	57	0	0.016	N/A
