# Supplementary material for: Identification of upstream miRNAs of SNAI2 and their influence on the metastasis of gastrointestinal stromal tumors
Source: Cancer Cell Int. 2019 Nov 12;19:289. doi: 10.1186/s12935-019-1006-8 (PMC6852720; doi:10.1186/s12935-019-1006-8)
Supplement: Supplementary file 2 — Additional file 2. The microarray profiling of miRNAs in high-SNAI2-level GISTs and low-SNAI2-level GISTs [file 12935_2019_1006_MOESM2_ESM.pdf]

Name	log2 (Ratio) H/L	Name	log2 (Ratio) H/L
hsa-miR-221-3p	4.96633957	hsa-miR-192-5p	-4.21418783
hsa-miR-222-3p	4.87149753	hsa-miR-133b	-3.91396843
hsa-miR-146b-5p	4.39099189	hsa-miR-133a-3p	-3.78889802
hsa-miR-181a-5p	4.12880165	hsa-miR-1-3p	-3.48465569
hsa-miR-585-5p	4.00926602	hsa-miR-194-5p	-3.2852683
hsa-miR-34a-5p	3.9504062	hsa-miR-200c-3p	-2.96640703
hsa-miR-196a-5p	3.92717587	hsa-miR-200a-3p	-2.88413069
hsa-miR-574-3p	3.8123513	hsa-miR-141-3p	-2.47709234
hsa-miR-3663-3p	3.70896294	hsa-miR-3923	-2.28133376
hsa-miR-6504-3p	3.34292466	hsa-miR-215-5p	-2.15533807
hsa-miR-466	3.34163001	hsa-miR-200b-3p	-2.14569966
hsa-miR-455-3p	3.28645875	hsa-miR-655-5p	-1.99371374
hsa-miR-223-3p	3.16753727	hsa-miR-5687	-1.9693032
hsa-miR-8064	3.14191003	hsa-miR-1909-3p	-1.78508313
hsa-miR-149-5p	3.13304788	hsa-miR-3152-3p	-1.66785551
hsa-miR-129-2-3	2.81179981	hsa-miR-3687	-1.65299357
hsa-miR-424-5p	2.71065291	hsa-miR-3912-5p	-1.64533512
hsa-miR-181b-5p	2.67849063	hsa-miR-202-3p	-1.6303544
hsa-miR-4687-5p	2.67669284	hsa-miR-1258	-1.61361725
hsa-miR-8485	2.66795701	hsa-miR-4665-5p	-1.5901987
hsa-miR-218-5p	2.59837308	hsa-miR-190b	-1.56687204
hsa-miR-483-3p	2.58118877	hsa-miR-378f	-1.54955717
hsa-miR-6805-3p	2.57585943	hsa-miR-182-5p	-1.52892847
hsa-miR-132-3p	2.5404057	hsa-miR-28-3p	-1.49321257
hsa-miR-4324	2.53055809	hsa-miR-422a	-1.47880895
hsa-miR-34b-5p	2.52767525	hsa-miR-378e	-1.42584425
hsa-miR-1248	2.46752827	hsa-miR-378a-3p	-1.38830587
hsa-miR-6836-3p	2.44884914	hsa-miR-1910-3p	-1.36907919
hsa-miR-125b-5p	2.38905319	hsa-miR-378h	-1.36528446
hsa-miR-125a-5p	2.38098643	hsa-miR-3115	-1.32716474
hsa-miR-335-5p	2.37291771	hsa-miR-378d	-1.32033308
hsa-miR-3182	2.36908788	hsa-miR-378g	-1.31973499
hsa-miR-29b-3p	2.33330646	hsa-miR-4437	-1.29328308
hsa-miR-139-5p	2.3110547	hsa-miR-3945	-1.27905463
hsa-miR-3064-3p	2.30909705	hsa-miR-6841-5p	-1.25633975
hsa-miR-877-3p	2.30021863	hsa-miR-6864-5p	-1.2410081
hsa-miR-3607-5p	2.28298086	hsa-miR-6823-5p	-1.19183069
hsa-miR-6072	2.27934961	hsa-miR-378c	-1.18929776
hsa-miR-6892-3p	2.2722033	hsa-miR-10b-5p	-1.18005487
hsa-miR-6756-3p	2.1949655	hsa-miR-338-3p	-1.12791748
hsa-miR-30a-5p	2.15158771	hsa-miR-125b-1-	-1.11451188
hsa-miR-3151-3p	2.12379234	hsa-miR-3195	-1.10451049
hsa-miR-210-5p	2.11117418	hsa-miR-6889-5p	-1.10307796
hsa-miR-21-3p	2.10453058	hsa-miR-363-3p	-1.09432738
hsa-miR-7113-3p	2.10402788	hsa-miR-296-3p	-1.0847065
hsa-miR-339-5p	2.08608601	hsa-miR-378i	-1.07412904
hsa-let-7i-5p	2.07424867	hsa-miR-429	-1.05012348
hsa-miR-6727-3p	2.06862181	hsa-miR-588	-1.03527548
hsa-miR-197-3p	2.06852552	hsa-miR-199b-5p	-1.02020328
hsa-miR-29a-3p	2.05534219	hsa-miR-4754	-1.01875009
hsa-miR-3607-3p	2.04091842	hsa-miR-30c-1-3	-1.01520376
hsa-miR-6775-3p	2.04015659	hsa-miR-4753-5p	-1.00137859

hsa-miR-4763-5p	2.0340185
hsa-miR-4664-5p	2.03131909
hsa-miR-107	2.00127253
hsa-miR-758-5p	1.98759498
hsa-miR-6792-3p	1.98551674
hsa-miR-1228-3p	1.9639583
hsa-miR-4707-3p	1.94184083
hsa-miR-766-3p	1.91668644
hsa-miR-1273g-3	1.90696997
hsa-miR-6858-3p	1.90588352
hsa-miR-30d-5p	1.88101022
hsa-miR-181c-5p	1.8779009
hsa-miR-503-5p	1.87240222
hsa-miR-7109-3p	1.86971687
hsa-miR-193b-3p	1.85366304
hsa-miR-6804-3p	1.83946496
hsa-miR-7108-3p	1.83661978
hsa-miR-675-3p	1.82861927
hsa-miR-451a	1.81286123
hsa-miR-29c-3p	1.80162609
hsa-miR-129-1-3	1.79981337
hsa-miR-301a-3p	1.79582472
hsa-miR-6809-3p	1.76353077
hsa-miR-4486	1.76348543
hsa-miR-6894-3p	1.75698304
hsa-miR-3184-3p	1.74902526
hsa-miR-4524a-5	1.74050358
hsa-miR-6867-3p	1.7307578
hsa-miR-1224-3p	1.72828506
hsa-miR-6754-3p	1.72263916
hsa-miR-4701-5p	1.71957462
hsa-miR-6747-3p	1.714697
hsa-miR-6743-3p	1.70203846
hsa-miR-100-5p	1.69171038
hsa-miR-320b	1.6904438
hsa-miR-6740-3p	1.69007746
hsa-miR-6877-3p	1.68940567
hsa-miR-664a-3p	1.68845234
hsa-miR-1234-3p	1.68674721
hsa-miR-103a-3p	1.68403174
hsa-miR-22-3p	1.68013892
hsa-miR-196b-3p	1.6735938
hsa-miR-5096	1.66695117
hsa-miR-4769-3p	1.65298603
hsa-miR-99a-5p	1.64405296
hsa-miR-1273h-3	1.64255219
hsa-miR-4695-3p	1.63165473
hsa-miR-6511a-3	1.63143774
hsa-miR-196b-5p	1.62712491
hsa-miR-4728-3p	1.62516882
hsa-miR-455-5p	1.62343665
hsa-miR-4646-3p	1.6190557
hsa-miR-1260a	1.61142933
hsa-miR-619-5p	1.60154711

hsa-miR-6822-5p	-0.99955498
hsa-miR-4489	-0.99726676
hsa-miR-3122	-0.990382
hsa-miR-2278	-0.98540003
hsa-miR-4770	-0.98324326
hsa-miR-4792	-0.96860647
hsa-miR-3934-5p	-0.95826473
hsa-miR-3179	-0.95656074
hsa-miR-375	-0.93801311
hsa-miR-3665	-0.93604305
hsa-miR-7113-5p	-0.92885124
hsa-miR-6847-5p	-0.92353616
hsa-miR-6878-5p	-0.9231844
hsa-miR-8063	-0.92022944
hsa-miR-32-3p	-0.91420092
hsa-miR-659-3p	-0.91288934
hsa-miR-3136-5p	-0.91212724
hsa-miR-6129	-0.90536313
hsa-miR-520g-5p	-0.89653668
hsa-miR-5090	-0.89497252
hsa-miR-4658	-0.894647
hsa-miR-490-5p	-0.89201732
hsa-miR-3185	-0.87879829
hsa-miR-2909	-0.87446912
hsa-miR-744-5p	-0.87107054
hsa-miR-138-2-3	-0.857981
hsa-miR-3186-3p	-0.8437443
hsa-miR-6881-5p	-0.83750453
hsa-miR-1229-5p	-0.82801757
hsa-miR-500a-3p	-0.82160541
hsa-miR-5191	-0.8214991
hsa-miR-3144-5p	-0.81933419
hsa-miR-193a-5p	-0.81863485

hsa-miR-1229-3p	1.60094786
hsa-miR-6731-3p	1.60065918
hsa-miR-6763-3p	1.59706454
hsa-miR-6845-3p	1.59319109
hsa-miR-146a-5p	1.58118086
hsa-miR-6819-3p	1.57573393
hsa-miR-140-3p	1.56998424
hsa-miR-6793-3p	1.56052828
hsa-miR-6862-3p	1.55918977
hsa-miR-3935	1.55876856
hsa-miR-6861-3p	1.55794035
hsa-miR-6812-3p	1.55027182
hsa-miR-4758-3p	1.54874231
hsa-miR-6784-3p	1.54536659
hsa-miR-324-5p	1.54337044
hsa-miR-210-3p	1.54040289
hsa-miR-99b-5p	1.53513277
hsa-miR-484	1.53000462
hsa-miR-1260b	1.52121735
hsa-miR-3135b	1.51983986
hsa-miR-6813-3p	1.5183399
hsa-miR-6801-3p	1.51097762
hsa-miR-6879-3p	1.50505821
hsa-miR-30a-3p	1.502111
hsa-miR-6887-3p	1.49350625
hsa-miR-6730-3p	1.49334418
hsa-miR-6834-3p	1.48389091
hsa-miR-3622a-3	1.47719542
hsa-miR-6873-3p	1.47540886
hsa-miR-4749-3p	1.46850574
hsa-miR-6872-3p	1.46679913
hsa-miR-191-5p	1.4645379
hsa-miR-320a	1.46162344
hsa-miR-6769a-3	1.46122649
hsa-miR-4722-3p	1.46108613
hsa-miR-424-3p	1.46033585
hsa-miR-181d-5p	1.45311231
hsa-miR-423-3p	1.45286685
hsa-miR-7111-3p	1.42461245
hsa-miR-3620-3p	1.42453126
hsa-miR-148b-3p	1.42256046
hsa-miR-3651	1.4150375
hsa-miR-6776-3p	1.4137211
hsa-miR-6750-3p	1.41260327
hsa-miR-634	1.41172589
hsa-miR-1281	1.41132799
hsa-let-7e-5p	1.40801483
hsa-miR-6732-3p	1.40744035
hsa-miR-3972	1.40047079
hsa-miR-6886-3p	1.39929095
hsa-miR-1976	1.3860928
hsa-miR-21-5p	1.37250681
hsa-miR-30c-5p	1.37036213
hsa-miR-129-5p	1.36235914

hsa-miR-6785-3p	1.36211569
hsa-miR-4323	1.35255935
hsa-miR-1273a	1.34799591
hsa-miR-6882-3p	1.3431979
hsa-miR-320d	1.34108281
hsa-miR-664b-3p	1.33948647
hsa-miR-4258	1.33895511
hsa-miR-6890-3p	1.33833757
hsa-miR-4723-3p	1.3380778
hsa-miR-2110	1.32894852
hsa-miR-3119	1.32894852
hsa-miR-4433b-5	1.32621967
hsa-miR-6865-3p	1.3257783
hsa-miR-664a-5p	1.31124167
hsa-miR-1470	1.30256277
hsa-miR-942-3p	1.29658697
hsa-miR-1250-3p	1.29083906
hsa-miR-6833-3p	1.27623917
hsa-miR-6810-3p	1.27131232
hsa-miR-7110-3p	1.27054416
hsa-miR-6823-3p	1.26858742
hsa-let-7g-5p	1.26475088
hsa-miR-532-5p	1.25296813
hsa-miR-6803-3p	1.25047655
hsa-miR-668-3p	1.25046474
hsa-miR-6780b-3	1.2446572
hsa-miR-3145-5p	1.24315654
hsa-let-7b-3p	1.23982702
hsa-miR-4284	1.23584413
hsa-miR-4713-5p	1.23536003
hsa-miR-1285-5p	1.23470568
hsa-miR-6798-3p	1.23470568
hsa-miR-6788-3p	1.2321737
hsa-miR-6880-3p	1.22873953
hsa-miR-6777-3p	1.22017319
hsa-miR-6716-3p	1.20163386
hsa-miR-486-5p	1.19963791
hsa-miR-92b-3p	1.19159229
hsa-miR-5196-3p	1.19119193
hsa-miR-6779-3p	1.18720969
hsa-miR-199a-5p	1.18672916
hsa-miR-6815-3p	1.18210599
hsa-miR-6893-3p	1.16763319
hsa-miR-328-3p	1.16677116
hsa-miR-6820-3p	1.164653
hsa-miR-106b-5p	1.16353216
hsa-miR-6722-5p	1.16227672
hsa-miR-1825	1.1621632
hsa-miR-4717-5p	1.14369346
hsa-miR-3653-5p	1.14345003
hsa-miR-6760-3p	1.13601023
hsa-miR-4290	1.13321322
hsa-miR-26b-5p	1.13222556
hsa-miR-6752-3p	1.12753324

hsa-miR-4685-3p	1.10909693
hsa-miR-16-5p	1.10618687
hsa-miR-532-3p	1.10469738
hsa-miR-6796-3p	1.10433666
hsa-miR-664b-5p	1.102928
hsa-miR-30b-5p	1.10028233
hsa-miR-30e-5p	1.09966645
hsa-miR-5194	1.09776274
hsa-miR-6761-3p	1.09708926
hsa-miR-4254	1.09636839
hsa-miR-3175	1.08712418
hsa-miR-4745-3p	1.08690807
hsa-miR-6765-3p	1.08623405
hsa-miR-6741-3p	1.08574003
hsa-miR-6797-3p	1.08569955
hsa-let-7b-5p	1.08430378
hsa-miR-508-5p	1.08161377
hsa-miR-6849-3p	1.07942691
hsa-let-7d-5p	1.07938691
hsa-miR-1225-3p	1.06774461
hsa-miR-144-3p	1.06354796
hsa-miR-3617-3p	1.06122286
hsa-miR-29c-5p	1.06080328
hsa-miR-4750-3p	1.05201931
hsa-miR-204-5p	1.04559687
hsa-miR-320c	1.0444872
hsa-miR-25-3p	1.04264434
hsa-miR-425-5p	1.04215862
hsa-miR-615-3p	1.04102727
hsa-miR-4641	1.038789
hsa-miR-6511b-3	1.03847415
hsa-miR-6729-3p	1.03533307
hsa-miR-6509-3p	1.03266748
hsa-miR-186-5p	1.03095538
hsa-miR-4725-5p	1.02844398
hsa-miR-5193	1.02725622
hsa-miR-4633-5p	1.02621304
hsa-miR-4700-3p	1.02504259
hsa-miR-1291	1.02272008
hsa-miR-6790-3p	1.02223187
hsa-miR-7106-3p	1.02120218
hsa-miR-5684	1.02106162
hsa-miR-497-5p	1.0194912
hsa-miR-142-3p	1.01856762
hsa-miR-5100	1.00880053
hsa-miR-4667-3p	1.00839592
hsa-miR-1296-5p	1.00749454
hsa-miR-4485-5p	1.00551357
hsa-miR-6746-3p	1.00444591
hsa-miR-6800-3p	1.00240249
hsa-miR-1285-3p	1.00069049
hsa-miR-6787-3p	1.0004834
hsa-miR-1322	0.99548012
hsa-miR-6769b-3	0.99518761

hsa-miR-1301-3p	0.99421765
hsa-miR-3907	0.98830528
hsa-miR-4313	0.9867439
hsa-miR-660-5p	0.9866622
hsa-miR-6875-3p	0.98429256
hsa-miR-6884-3p	0.98284842
hsa-miR-1249-3p	0.97978678
hsa-miR-93-5p	0.97966165
hsa-let-7a-3p	0.97780425
hsa-miR-1193	0.97741634
hsa-miR-7114-3p	0.97130112
hsa-miR-6728-3p	0.96413455
hsa-miR-6782-3p	0.95935802
hsa-miR-1233-3p	0.956456
hsa-let-7a-5p	0.95221633
hsa-miR-6759-3p	0.95087477
hsa-miR-185-5p	0.95047678
hsa-let-7f-5p	0.94655784
hsa-miR-6508-5p	0.94263378
hsa-miR-1914-3p	0.94147805
hsa-miR-6859-3p	0.93451092
hsa-miR-4433a-5	0.93444746
hsa-miR-3944-3p	0.93214291
hsa-miR-6737-3p	0.92599942
hsa-miR-551b-3p	0.92283214
hsa-miR-1292-3p	0.92199749
hsa-miR-320e	0.91659932
hsa-miR-5010-5p	0.91555387
hsa-miR-151a-3p	0.90941215
hsa-miR-6772-3p	0.90372112
hsa-miR-148a-3p	0.90346445
hsa-miR-4448	0.9015543
hsa-miR-330-3p	0.90092023
hsa-miR-4638-5p	0.89957253
hsa-miR-324-3p	0.89897008
hsa-miR-6736-3p	0.89879366
hsa-miR-339-3p	0.89251557
hsa-miR-6870-3p	0.89049582
hsa-miR-92a-3p	0.88963456
hsa-miR-31-5p	0.88820918
hsa-miR-196a-3p	0.88820918
hsa-miR-26a-5p	0.88702402
hsa-miR-5095	0.88625901
hsa-miR-29b-2-5	0.8799693
hsa-miR-6883-3p	0.87918359
hsa-miR-15a-5p	0.87887165
hsa-miR-939-3p	0.87788887
hsa-miR-6130	0.87219555
hsa-miR-6734-3p	0.86821013
hsa-miR-6514-3p	0.86577161
hsa-let-7c-5p	0.8635441
hsa-miR-361-5p	0.86263343
hsa-miR-363-5p	0.86040672
hsa-miR-3609	0.86038638

hsa-miR-6717-5p	0.84991538
hsa-miR-146b-3p	0.84950837
hsa-miR-550b-2-	0.84890741
hsa-miR-6802-3p	0.83984816
hsa-miR-342-3p	0.83920379
hsa-miR-6832-3p	0.83748879
hsa-miR-143-5p	0.83653578
hsa-miR-34c-5p	0.83650127
hsa-miR-4524b-5	0.8353693
hsa-miR-1273e	0.83279751
hsa-miR-6786-3p	0.82823365
hsa-miR-6831-3p	0.82759571
hsa-miR-6773-3p	0.82269547
hsa-miR-874-5p	0.81690685
hsa-miR-6825-3p	0.81068348
hsa-miR-3189-5p	0.80385748
hsa-miR-4787-3p	0.80240004
